# Nitrogen Management in Crop–Soil–Environment Systems: Pathways Toward Sustainable and Climate-Resilient Agriculture

**DOI:** 10.3390/ijms27052477

**Published:** 2026-03-08

**Authors:** Szilvia Veres, Nevien Elhawat, Zed Rengel, Tarek Alshaal

**Affiliations:** 1 Institute of Applied Plant Biology, Faculty of Agricultural and Food Sciences and Environmental Management, University of Debrecen, 4032 Debrecen, Hungary; szveres@agr.unideb.hu; 2 Department of Food Biotechnology, Albert Kazmer Mosonmagyarovar Faculty, Széchenyi István University, 9026 Győr, Hungary; 3 Faculty of Agriculture (for Girls), Al-Azhar University, Cairo 11884, Egypt; 4 UWA School of Agriculture and Environment, University of Western Australia, Perth, WA 6009, Australia; zed.rengel@uwa.edu.au; 5 Institute for Adriatic Crops and Karst Reclamation, 21000 Split, Croatia; 6 Soil and Water Science Department, Faculty of Agriculture, Kafrelsheikh University, Kafr El-Sheikh 33516, Egypt

**Keywords:** nitrogen cycle, circular nitrogen economy, precision agriculture, climate-resilient strategies

## Abstract

Abiotic stresses including drought, salinity, heat, cold, and heavy metal toxicity severely constrain plant productivity worldwide. Nitrogen (N), beyond its fundamental nutritional role, has emerged as a central regulator of plant stress responses through its involvement in metabolic reprogramming, osmotic adjustment, antioxidant defense, and hormonal signaling. This review synthesizes current advances in understanding how nitrogen availability and form influence plant tolerance to major abiotic stresses. Particular emphasis is placed on nitrogen-mediated modulation of reactive oxygen species (ROS) scavenging systems, nitrogen–carbon metabolic coordination, phytohormonal crosstalk, osmoprotectant biosynthesis, and regulation of stress-responsive gene expression. Recent molecular insights highlight the role of nitrogen transporters, nitrate signaling pathways, and nitrogen-use efficiency in stress adaptation mechanisms. Furthermore, agronomic and biotechnological strategies aimed at optimizing nitrogen management to enhance stress resilience are discussed, including precision fertilization, integrated nutrient management, and genetic approaches targeting nitrogen-responsive regulatory networks. By integrating physiological, biochemical, and molecular perspectives, this review provides a comprehensive framework for understanding nitrogen-driven mitigation strategies under abiotic stress conditions and outlines future research directions for sustainable crop production in changing environments.

## 1. Introduction

Nitrogen (N) is a cornerstone of life on Earth, playing an indispensable role in plant metabolism as a building block of amino acids, nucleotides, and chlorophyll, which are essential for photosynthesis and growth. In agricultural systems, nitrogen availability is a critical determinant of crop productivity, directly influencing global food security. However, nitrogen use efficiency (NUE) in these systems is alarmingly low, with only 30–50% of applied nitrogen fertilisers typically utilized by crops, while the remainder is lost through processes such as volatilization, leaching, and denitrification [1]. These losses have profound environmental consequences, contributing to eutrophication of water bodies, soil acidification, and the release of nitrous oxide (N_2_O), a greenhouse gas with a global warming potential nearly 300 times that of CO_2_ [2].

The dynamics of nitrogen in the soil are governed by intricate biological and chemical interactions. Microorganisms play a pivotal role in nitrogen cycling, facilitating processes such as nitrification, denitrification, and biological nitrogen fixation. However, intensive agricultural practices and heavy reliance on synthetic fertilisers have disrupted these natural cycles [3]. In response, researchers are exploring innovative strategies to enhance nitrogen use efficiency and reduce the environmental footprint of nitrogen use in agriculture. Recent advancements include the development of slow- and controlled-release fertilisers that minimize nitrogen losses; microbial inoculants that enhance nitrogen fixation and assimilation; and precision agriculture technologies, such as soil sensors and satellite imaging, that enable site-specific nutrient management [4].

Furthermore, understanding the physiological mechanisms of nitrogen uptake, assimilation, and remobilization in plants has opened new avenues for crop improvement. Advances in genomics and gene-editing technologies, such as CRISPR-Cas9, hold promise for developing crop varieties with enhanced nitrogen uptake efficiency (NUpE) by optimizing root architecture, transport systems, and metabolic pathways, though such applications remain largely in the research and proof-of-concept stages [5]. Integrating these scientific insights with sustainable practices, such as combining organic and inorganic nitrogen sources, can promote a circular nitrogen economy, reducing dependency on synthetic fertilisers and mitigating nitrogen losses. As climate change exacerbates nitrogen-related challenges, particularly in terms of altered nitrogen cycling and increased greenhouse gas emissions, there is an urgent need for adaptive nitrogen management strategies [6].

This review provides a comprehensive exploration of the role of nitrogen in plant, soil, and environmental systems, focusing on innovative approaches to improve nitrogen use efficiency and address the environmental impacts of nitrogen mismanagement. By synthesizing recent research and highlighting interdisciplinary solutions, it aims to inform sustainable nitrogen management practices that align with global efforts to achieve food security and mitigate climate change.

## 2. Nitrogen: The Essential Element in Plant and Soil Dynamics

### 2.1. Nitrogen in Plant Metabolism and Growth

Nitrogen plays a pivotal role in plant health and development, being indispensable for the synthesis of amino acids (the building blocks of proteins) and nucleic acids (a core component of DNA and RNA) [7]. Nitrogen constitutes 10–50 g/kg of plant dry matter [7]. Although nitrogen is abundant in the atmosphere, comprising about 78% of the air, plants cannot directly utilize this inert form (N_2_). It must first undergo fixation, a process facilitated by industrial methods such as the Haber–Bosch process or through microbial activity [8,9]. In soil, nitrogen is present in various forms, including inorganic ionic forms like nitrate (NO_3^−^_) and ammonium (NH_4^+^_), and organic forms such as urea, free amino acids, and peptides. However, plant capacity to access these forms varies depending on microbial transformations, soil heterogeneity, and environmental factors [10]. Nitrogen availability is crucial for photosynthesis [11], plant metabolism, seed germination, hormonal balance, and stress resistance. Proper nitrogen utilsation enhances root development, leading to improved water absorption and nutrient uptake [8]. However, the global reliance on nitrogen fertilisers to boost crop productivity has led to environmental and socioeconomic challenges, emphasizing the need for more sustainable practices [12]. Recent studies underscore the importance of understanding nitrogen nutrition as a complex process, accounting for the interactions between its various forms and their effects on plant growth and metabolism [9,13,14]. The integration of “omics” approaches offers promising insights into optimizing nitrogen use efficiency while minimizing its ecological footprint.

Nitrogen use efficiency is a composite indicator describing how effectively nitrogen inputs are converted into economic yield and is most commonly defined as the ratio of crop yield to the amount of nitrogen supplied or available in the soil system [15,16].

$$NUE=\frac{Economic\;yield}{Nitrogen\;supplied\;or\;available}$$

Conceptually, NUE can be partitioned into two physiological components: nitrogen uptake efficiency (NUpE) and nitrogen utilisation efficiency (NUtE). Nitrogen uptake efficiency (NUpE) quantifies the plant’s capacity to acquire nitrogen from the soil and is calculated as the ratio of total plant nitrogen uptake to the nitrogen supplied or available, reflecting root traits, transporter activity, and rhizosphere processes [12,15].

$$NUpE=\frac{Total\;plant\;nitrogen\;uptake}{Nitrogen\;available\;in\;soil\;or\;applied}$$

Nitrogen utilisation efficiency (NUtE) describes the efficiency with which absorbed nitrogen is converted into yield and is calculated as yield per unit of plant nitrogen uptake, integrating internal nitrogen assimilation, remobilization, and allocation processes [17].

$$NUtE=\frac{Yield\;or\;biomass}{Total\;plant\;nitrogen\;uptake}$$

Under this framework, overall nitrogen use efficiency is mathematically expressed as the product of NUpE and NUtE rather than their sum [15].

(1) NUE=NUpE×NUtE

In contrast, agronomic efficiency (AE) is a management-oriented metric that quantifies the yield response per unit of applied fertiliser nitrogen and is calculated as the yield difference between fertilized and unfertilized treatments divided by the amount of nitrogen applied [18].

(2) $$AE=\frac{{Yield}_{fertilised}{-Yield}_{unfertilised}}{Amount\;of\;nitrogen\;applied}$$

The agronomic efficiency integrates both soil nitrogen recovery and plant utilisation processes but is not a physiological component of nitrogen use efficiency; instead, it reflects fertiliser management effectiveness under specific environmental conditions [16].

**Figure 1:**
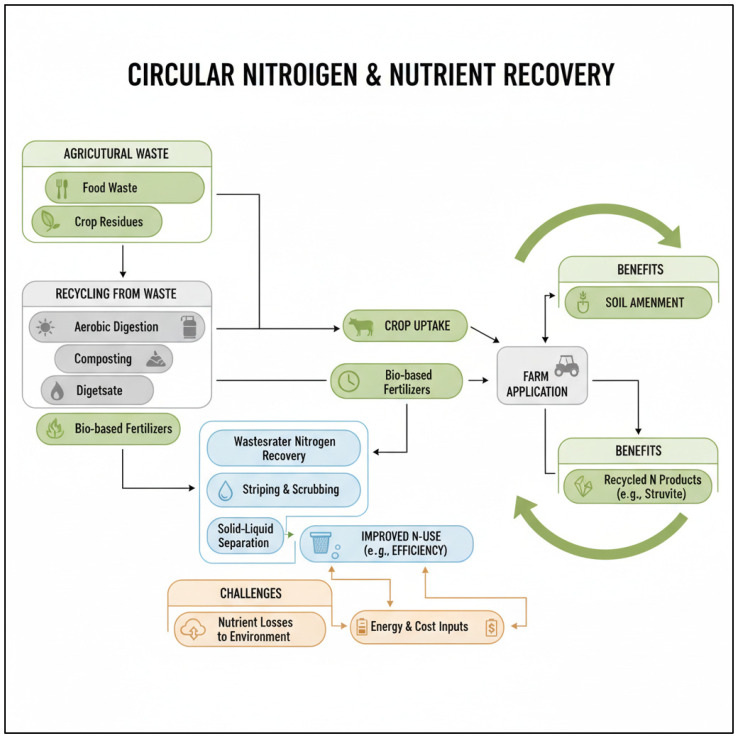
Flowchart of cutting-edge strategies for enhancing nitrogen use efficiency (NUE) with outcomes and implementation challenges.

The integrated processes governing nitrogen uptake, assimilation, and internal redistribution that underpin nitrogen use efficiency are illustrated in Figure 1. The figure highlights key control points—including transporter activity, metabolic assimilation, and source–sink allocation—that are frequently targeted by agronomic, genetic, and biotechnological strategies to improve NUE. By visualizing how inorganic and organic nitrogen forms are acquired and integrated into plant metabolism, Figure 1 provides a mechanistic foundation for understanding how interventions such as crop breeding, genome editing, optimized fertilization, and bio-based inputs can enhance nitrogen capture, utilization, and retention within plant–soil systems.

### 2.2. Nitrogen Forms, Nitrogen Uptake, and Transport Mechanisms in Plants

The accessibility of different nitrogen forms to plants is significantly influenced by intricate microbial transformations and spatial and temporal heterogeneity of the soil physical, chemical, and biological properties, which collectively govern the efficiency of nitrogen uptake and plant growth [19]. Three primary protein classes involved in nitrogen uptake include transporters, receptors, and transceptors. Transporters mediate the selective transport of nitrogen, whereas transceptors combine transport and sensing functions [20]. Post-translational modifications, particularly phosphorylation, are essential for modulating transporter activity, allowing plants to respond rapidly to changes in nitrogen availability [21,22]. The nitrogen concentration within root cells is influenced by several factors, including external nitrogen availability, net uptake (influx minus efflux), root metabolism, vacuolar storage, and transport system [23].

#### 2.2.1. Nitrate Uptake

Nitrate is the predominant nitrogen source in most agricultural soils. Its uptake relies on symport systems with protons (2H^+^/NO_3^−^_) [24]. The uptake of NO_3^−^_ in plants is mediated by two major families of transporters, i.e., NPF (formerly NRT1/PTR) and NRT2 [25]. These transporters are characterized by 12 transmembrane domains and are tightly regulated by NO_3^−^_ availability and the plant nitrogen nutritional status [26].

In arabidopsis, NRT1.1 (also known as CHL1) is a key transceptor for NO_3^−^_ uptake, functioning in both low-affinity (K_m_ ~4 mM) and high-affinity (K_m_ ~50 µM) modes, depending on the phosphorylation state of threonine-101. This phosphorylation, controlled by the CIPK23 kinase, is crucial for adapting to varying NO_3^−^_ levels [27]. Furthermore, NRT1.1 plays a role as a NO_3^−^_ sensor, influencing root system architecture and modulating NO_3^−^_ transporter expression [22]. The NRT2 family, which includes NRT2.1 and NRT2.2, is primarily responsible for high-affinity NO_3^−^_ uptake (<0.25 mM). The NRT2 proteins cooperate with NAR2.1 to form stable complexes for efficient NO_3^−^_ transport. Post-translational modifications, such as phosphorylation at serine-28 and serine-510 in NRT2.1, regulate its activity in response to changing NO_3^−^_ supplies [28].

Even though arabidopsis serves as a model for studying nitrogen transport, similar mechanisms in crops must be considered with caution. For instance, maize expresses ZmNPF6.6, a homolog of NRT1.1, that shares some regulatory features but shows distinct kinetic and functional differences [29]. In rice (*Oryza sativa*), a model for cereal crops, nitrogen transport is a sophisticated system critical for agronomic yield and nitrogen use efficiency. Nitrate acquisition and signaling are managed by distinct transporter families; the low-affinity transporter OsNRT1.1b (OsNPF6.5b) has been identified as a pivotal quantitative trait locus enhancing NUE and grain yield [30], while the high-affinity system requires the formation of a heteromeric complex between OsNRT2.1/2.2 and their partner protein OsNAR2.1 for functional transport [31]. Subsequent internal translocation of assimilated N to the grain is mediated by amino acid transporters, with OsAAP6 playing a crucial role in regulating grain protein content and overall yield by facilitating phloem loading of amino acids [32].

Advances in proteomics and molecular studies are unveiling new regulatory mechanisms that control nitrogen uptake, particularly through nitrate transporters, providing pathways to improve nitrogen uptake efficiency (NUpE) and sustainability in agriculture. In rice, for instance, high-throughput proteomic analyses have identified post-translational modifications, such as phosphorylation, that modulate the activity and localization of nitrate transporters like OsNRT2.1 and OsNRT2.3a, enabling rapid responses to fluctuating soil nitrate levels and enhancing root uptake under nitrogen-limiting conditions. Molecular investigations, including transcriptomics and CRISPR-based gene editing, reveal that OsNPF6.3 (OsNRT1.1B) not only facilitates nitrate influx but also acts as a nitrate sensor triggering downstream signaling cascades involving OsNLP3 transcription factors, which reprogram nitrogen assimilation pathways to optimize growth and reduce fertiliser runoff. Similarly, in maize, ZmNPF6.6 phosphorylation by the ZmCIPK23 kinase regulates nitrate transport affinity, whereas in arabidopsis, AtNRT1.1 (CHL1) undergoes dynamic ubiquitination influencing its endosymbiotic interactions and auxin crosstalk for root architecture remodeling. These insights pave the way for breeding nitrate-efficient crop varieties via targeted genetic interventions, minimizing environmental impacts such as eutrophication [33,34,35,36].

#### 2.2.2. Ammonium Uptake and Regulation

Ammonium is a key nitrogen source affecting plant growth and root system architecture. While efficiently absorbed and assimilated, excessive NH_4^+^_ leads to toxicity, causing stunted growth, chlorosis, and poor root development. A balanced ratio of NO_3^−^_ to NH_4^+^_ is essential for optimal growth, varying by species, growth stages, and conditions [37]. NH_4^+^_ uptake depends on the plant nutritional status. Cytosolic NH_4^+^_ levels range from 1 to 10 mM, vacuolar levels from 1 to 45 mM, and root apoplast levels are consistently buffered at 1–2 mM. At high external concentrations, NH_4^+^_ enters cells as NH_3_ via aquaporins or as NH_4^+^_ through non-selective cation and potassium channels, such as AKT1 [38]. At low NH_4^+^_ concentrations (<1 mM), AMT1 proteins (ammonium transporter 1) regulate uptake, functioning as NH_4^+^_-uniporters or NH_3_/H^+^ co-transporters. In arabidopsis, AMT1;1, AMT1;3, and AMT1;5 work additively in root epidermis and hairs. The AMT1;5 has high affinity (K_m_ ~5 µM) for NH_4^+^_ during nitrogen starvation, whereas AMT1;2, with lower affinity (K_m_ ~230 µM), retrieves NH_4^+^_ from the apoplast. The AMT1 expression is influenced by nitrogen starvation, diurnal cycles, and metabolites like glutamine [39].

Phosphorylation at the C-termini regulates AMT1 proteins, preventing toxicity. For example, threonine-460 phosphorylation inactivates AMT1;1 [39]. Additional phosphorylation sites, such as threonine-464 and threonine-494 in AMT1;3, influence activity and induce trans-inactivation when forming complexes with AMT1;1 [40]. Even though NH_4^+^_ uptake mechanisms are conserved, crops show differences. In maize, NH_4^+^_ influx initially increases upon supply but is inhibited after hours by local NH_4^+^_ signals, independent of the plant nutritional status [41]. These differences highlight the need for research into crop-specific NH_4^+^_ nutrition to understand their sensitivity and adaptability.

#### 2.2.3. Amino Acid Uptake and Transport

Amino acids range in concentration from 1 to 10 mM in plant tissues and can vary significantly under high NH_4^+^_ nutrition. Major amino acids like glutamate, glutamine, aspartate, asparagine, and alanine dominate, while minor ones are tightly regulated [21]. In soils, amino acid concentrations range from 1 to 150 µM, influenced by environmental factors, microbial activity, and soil properties, which limit the mobility and availability of essential amino acids like arginine and lysine. Although plants can take up amino acids from soil, their effectiveness as nitrogen sources remains debated due to their low diffusion rates and short half-life in the soil [42].

In hydroponics, amino acid supply generally yields positive results, but soil studies often show limited contributions, possibly due to methodological biases. Specific high-affinity amino acid transporters in arabidopsis, such as LHT1, AAP1, and AAP5, mediate amino acid uptake and distribution across tissues. These transporters, which are part of the amino acid/auxin permease (AAAP) family, function as H^+^/amino acid symporters and exhibit tissue-specific expression. The LHT1 facilitates the uptake of neutral, acidic, and basic amino acids in roots and leaves, whereas AAP5, found primarily in roots, mediates the transport of lysine and arginine. The amino acid permease (AAP1) and proline transporter 2 (ProT2) handle the uptake of amino acids like alanine, glutamine, proline, and serine, with ProT2 also implicated in stress responses. The roles of these transporters evolve with plant age, with LHT1 and AAP5 primarily facilitating root uptake during early growth stages, and later shifting toward aerial transport functions as the plant matures [43]. Transporter gene regulation is complex, influenced by amino acid availability, light, stress conditions, and other nitrogen sources like NO_3^−^_ and NH_4^+^_, reflecting their dynamic roles in nitrogen nutrition and broader plant development [44].

The physiological capacity of plants to take up and utilise organic nitrogen forms, including amino acids, provides an important mechanistic foundation for circular nitrogen management strategies. Although the quantitative contribution of direct amino acid uptake to total plant nitrogen acquisition in mineral soils is often limited under conventional conditions, this pathway becomes increasingly relevant in systems enriched with organic inputs, such as composts, digestates, protein hydrolysates, and other bio-based fertilisers derived from agricultural, food, or industrial waste streams [45,46]. In such systems, microbial turnover and partial mineralisation can generate localised zones with elevated amino acid availability in the rhizosphere, where high-affinity transporters (e.g., LHT1, AAP family members) may facilitate direct uptake before complete conversion to inorganic nitrogen [47].

This physiological capability links plant nitrogen nutrition directly to circular economy concepts, in which nitrogen recovered from organic residues is returned to cropping systems in biologically compatible forms. Rather than relying exclusively on mineralization to nitrate or ammonium, bio-based fertilisers may exploit both microbial and plant-driven nitrogen acquisition pathways, potentially improving nitrogen retention, reducing leaching losses, and enhancing synchronization between nitrogen release and plant demand [48,49]. Consequently, understanding amino acid transport and regulation is not only of fundamental interest but also critical for optimizing the design, formulation, and application strategies of recycled organic fertilisers within circular nitrogen frameworks.

#### 2.2.4. Urea Uptake

Urea fertilisers account for over 50% of global nitrogen applications due to their low cost and high nitrogen content. In soils, microbial urease hydrolyzes urea into NH_4^+^_, later converted to NO_3^−^_ by nitrifying bacteria. Even though urea concentrations in soil are typically low (up to 70 µM), urea is now recognized as a direct nitrogen source for plants, supported by the discovery of specific high-affinity urea transporters [50]. Additionally, urea plays a key role in nitrogen recycling within plants, where it is metabolized by endogenous urease to release NH_4^+^_ for re-assimilation [9].

The DUR3, the first identified urea transporter in higher plants, is a high-affinity urea/H^+^ symporter in arabidopsis. Located in the plasma membrane of roots, DUR3 sustains up to 90% of urea uptake in the high-affinity range (K_m_ ~4 µM). Low-affinity uptake is mediated by transport through aquaporins [51]. Urea uptake is metabolically less efficient than inorganic nitrogen forms, but its provision as the sole nitrogen source induces the expression of AtDUR3 and the genes involved in amino acid metabolism and transport [52]. The AtDUR3 gene is highly expressed during nitrogen starvation, further induced by urea resupply, but suppressed by NH_4^+^_ or NO_3^−^_ resupply. Expression is strongest in the root epidermis and cortex, with moderate levels in vascular tissues near the xylem. Transcriptomic and physiological studies confirm that urea uptake is substrate-stimulated and reduced in the presence of inorganic nitrogen [52].

Studies in maize and wheat show that combining urea with NO_3^−^_ enhances plant growth and nitrogen uptake efficiency by reprogramming metabolic pathways, achieving a better balance. This effect mirrors findings from co-provision of NO_3^−^_ and NH_4^+^_, linked to changes in enzymes involved in carbon and nitrogen metabolism, water balance, and stress responses [53]. The DUR3 orthologues are present in various crops, with studies confirming their role in root urea uptake and suggesting a function in vascular urea loading in leaves [54]. Variations in DUR3 transcript abundance among tomato cultivars with differing nitrogen utilisation efficiency highlight its relevance [55]. Proteomic analysis in arabidopsis found DUR3 abundance higher under NH_4^+^_-adapted conditions, with phosphorylation at serine-568 influenced by NH_4^+^_ depletion, though its functional impact is not yet clear [56].

## 3. Nitrogen Cycling in Soil: Biological and Chemical Perspectives

**Figure 2:**
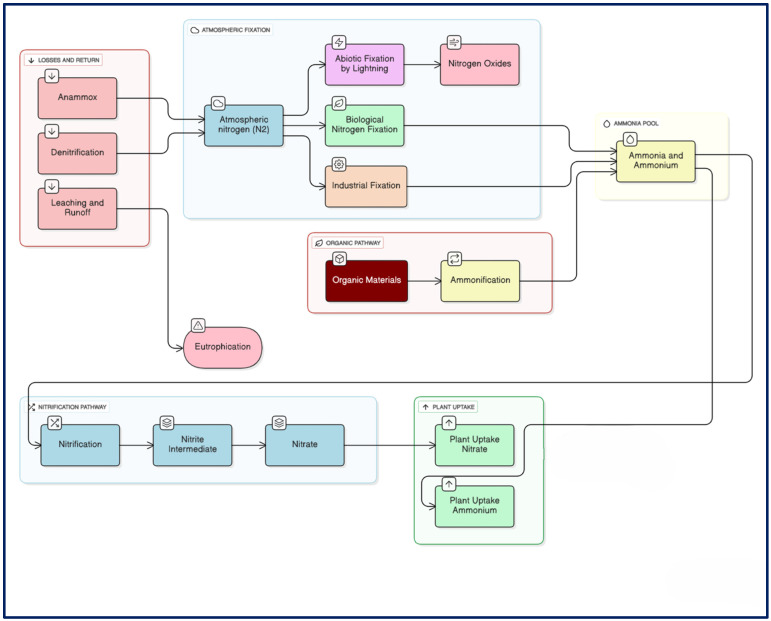
Flowchart illustrating the nitrogen cycle in soil, depicting biological processes (e.g., nitrogen fixation and nitrification) and losses (e.g., leaching, surface run-off, and denitrification).

The nitrogen cycle features a series of processes in which nitrogen transitions between living organisms and non-living components, such as the atmosphere, soil, and water (Figure 2). To move between different parts of the cycle, nitrogen transforms into various chemical forms. In the atmosphere, nitrogen primarily exists as gas (N_2_). In soil, it can be found as nitrogen oxide (NO), nitrogen dioxide (NO_2_), NH_4^+^_, and nitrate [57].

The nitrogen cycle consists of six main stages: fixation, volatilization, ammonification, nitrification, immobilization, and denitrification (Figure 2). During fixation, nitrogen gas in the atmosphere is converted into volatile NH_3_ by soil microbes. Another important aspect of the cycle is leaching, where NO_3^−^_ dissolves in water and drains from the soil, which can lead to contamination of nearby water sources or groundwater. These processes determine how nitrogen is available to plants and how it affects soil fertility [58].

### 3.1. Nitrogen Fixation

Nitrogen fixation is the conversion of atmospheric nitrogen into NH_3_/NH_4^+^_, a form usable by plants. This process can be biological or abiotic.

#### 3.1.1. Abiotic Nitrogen Fixation

Nitrogen predominantly exists as N≡N. This triple bond is exceptionally strong, with dissociation energy of 945 kJ/mol at 298 K, ranking it among the sturdiest covalent bonds and necessitating significant energy to cleave [59].

Human intervention in the nitrogen cycle began significantly with agriculture. The development of the Haber–Bosch process in 1913 revolutionized synthetic nitrogen fixation, enabling large-scale NH_3_ production and dramatically boosting agricultural productivity. Currently, over 500 million tons of NH_3_ are produced industrially each year [60].

The development of the Haber–Bosch process in the early 20th century revolutionized synthetic nitrogen fixation by enabling large-scale ammonia production from atmospheric nitrogen. This technological breakthrough fundamentally transformed global agriculture, as industrial ammonia synthesis became the backbone of nitrogen fertiliser production, supporting nearly half of global food production [61].

However, the process is highly energy-intensive and relies predominantly on fossil fuel–derived hydrogen, contributing substantially to global CO_2_ emissions. As a result, improving nitrogen use efficiency and exploring alternative, low-carbon nitrogen fixation technologies remain central priorities in sustainable nitrogen management and climate-resilient agricultural systems [62,63].

Research into alternative, sustainable methods for NH_3_ synthesis began decades ago. In the 1970s, Schrauzer and Guth used UV light to excite titania (TiO_2_), successfully reducing nitrogen gas with water to produce NH_3_ [64]. Later studies confirmed that TiO_2_, supplemented doped with low concentrations of Fe (a key element in the Haber–Bosch process) could achieve photocatalytic NH_3_ synthesis. Other semiconductors, including FeTiO_3_ [65], ZnO, CdS, SrTiO_3_, and GaP, alone or combined with platinum black (powder form of platinum), were explored, resulting in improved NH_3_ synthesis.

The first reported electrochemical synthesis of NH_3_ from nitrogen gas was conducted by Garagounis et al. [66] using Fe-based cathodic electrocatalysts with water as a proton source. Despite these pioneering efforts, significant interest in sustainable NH_3_ synthesis only emerged in the 21st century, driven by the urgency to address carbon emissions and prioritize sustainability. Several reviews have explored nitrogen fixation and NH_3_ production, focusing on plasma catalysts [67], electrocatalysts [68], and photocatalysts [69]. However, a comprehensive understanding of electron-driven NH_3_ production and catalyst design remains limited. Recent advances in atmospheric nitrogen fixation for NH_3_ synthesis include using renewable energy sources [70].

#### 3.1.2. Biological Nitrogen Fixation

Biological nitrogen fixation (BNF) represents around two-thirds of the global nitrogen fixation. This process occurs through both symbiotic and non-symbiotic associations between diazotrophic microorganisms and plants [71]. In symbiotic systems, nitrogen-fixing rhizobia (e.g., *Rhizobium* sp.) form root nodules on legumes and certain non-legumes, enabling mutualistic BNF that supplies host plants with fixed nitrogen [72]. In contrast, free-living plant growth-promoting rhizobacteria (PGPR) and other non-symbiotic diazotrophs, such as *Azoarcus* sp., *Beijerinckia* sp., *Pantoea agglomerans*, and *Klebsiella pneumoniae*, fix atmospheric nitrogen in soils independently of nodule formation, often associating loosely with plant roots [72] (Table 1). In nature, reactive nitrogen forms (those usable by living organisms) are synthesized primarily from atmospheric nitrogen by bacteria that produce NH_3_. This process, mediated by nitrogenase enzymes, is highly complex and varies across different bacteria. The FeMo nitrogenase, for instance, requires 8 ATP molecules per molecule of NH_3_ synthesized, with a total energy input of 244 kJ/mol. The general reaction for nitrogen fixation is as follows [73]:

N_2_ + 8e^−^ + 8H^+^ + 16ATP → 2NH_3_ + H_2_ + 16ADP + 16P_i_

The nitrogenase enzyme complex is encoded by a suite of nif genes (e.g., nifH, nifD, nifK for structural components, and others like nifA, nifL for regulation). These genes facilitate the activation of the iron protein (NifH), electron transfer, synthesis of the iron-molybdenum cofactor (FeMo-co), and various regulatory mechanisms essential for overall enzyme assembly and activity [74].

**Table 1:** Role of plant growth-promoting rhizobacteria (PGPR) in enhancing plant growth and development in recent studies.

PGPR	Crop	Impacts	Mechanism	References
*Azospirillum brasilense*	Maize (*Zea mays* L.)	Seed inoculation with *A. brasilense* Ab-V5 enhanced plant growth, boosted biochemical characteristics, and improved nitrogen use efficiency under nitrogen deficit conditions.	Seed inoculation with *A. brasilense* Ab-V5 → early IAA increase → rapid root system expansion → improved water & nutrient uptake → enhanced biochemical resilience → better N assimilation & partial N_2_ fixation → higher biomass & NUE under low N.	[75]
*Azospirillum brasilense* and *Bacillus subtilis*	Wheat (*Triticum aestivum* L.)	Seed inoculation with *A. brasilense* and *B. subtilis* enhanced grain nitrogen accumulation, increased the number of productive tillers per meter, grains per spike, and grain yield in irrigated wheat grown in tropical savannah conditions, regardless of nitrogen fertiliser application rates.	Seed inoculation → early root stimulation (IAA, ACC deaminase) → larger and more efficient root system → improved N uptake & assimilation + partial N fixation → enhanced tillering and spike fertility → higher grain N accumulation and grain yield, regardless of N fertilizer rate.	[76]
*Pseudomonas* strains P61, A46, JLB4	Blackberries (*Rubus* spp.)	Strain P61 increased plant height by 50% over the control. This is attributed to the increased production of phytohormones (auxins).	Strain P61 → high auxin production → increased stem cell elongation + improved root architecture → enhanced nutrient uptake → accelerated vegetative growth → ~50% increase in plant height.	[77]
Various genera (i.e., *Azoarcus*, *Azospirillum*, *Bacillus*, *Pseudomonas*)	Multiple crops: rice (*Oryza sativa* L.), wheat, sugarcane (*Saccharum officinarum*)	PGPR enhanced plant growth through nitrogen fixation, phytohormone production (auxins, cytokinins, gibberellins), phosphate solubilization, siderophore production, and induction of plant stress resistance.	PGPR colonize the rhizosphere → fix nitrogen + solubilize phosphorus + produce siderophores → improve nutrient uptake → release auxins, cytokinins, gibberellins → stimulate root and shoot growth → activate stress-resistance pathways → enhanced biomass, vigor, and yield.	[78]
*Bacillus* spp. and *Pseudomonas* spp.	Brazilian firetree (*Schizolobium parahyba*)	PGPR application improved early plant development (root and stem).	PGPR colonize the rhizosphere → release auxins, cytokinins, gibberellins → stimulate root and shoot cell division and elongation → solubilize nutrients and improve uptake → enhance stress resilience → faster and stronger early root and stem development.	[79]
Various PGPR strains	Multiple crops	PGPR contributed to sustainable agriculture by enhancing nutrient uptake and reducing reliance on synthetic fertilisers.	PGPR colonize the rhizosphere → release auxins, cytokinins, gibberellins → stimulate root and shoot cell division and elongation → solubilize nutrients and	[80]
*Burkholderia pyrrocinia* (FJS-3), *Pseudomonas rhodesiae* (FJS-7), *Pseudomonas baetica* (FJS-16)	Tea (*Camellia sinensis*), tobacco (*Nicotiana tabacum*), chili pepper (*Capsicum annuum*)	Increased new shoots in tea seedlings. In tobacco: FJS-3 most effective; multi-strain increased height 30.15%, fresh weight 37.36%, root weight 54.5%. In pepper: multi-strain increased height 30.10%, fresh weight 56.38%, root weight 43.18% vs. single strain. Field trial (Longjing43 tea): PGPR + fertiliser (T2) increased yield 15.38% vs. fertiliser alone; PGPR alone (T3) increased yield 92.31% vs. no input. Enhanced tea polyphenols, caffeine, theanine, and chlorophyll (improved Matcha color).	PGPR colonize the rhizosphere and root surfaces → secrete auxins, cytokinins, and gibberellins that accelerate shoot initiation, stem elongation, and root branching → solubilize phosphorus and mobilize nitrogen to increase nutrient availability during early growth → produce siderophores that enhance iron uptake and stimulate chlorophyll biosynthesis → expand root surface area and improve nutrient and water uptake efficiency → activate antioxidant and stress resilience pathways that stabilize growth under variable field conditions → increase new shoot formation in tea seedlings and boost height, fresh weight, and root biomass in tobacco and pepper → enhance tea leaf biochemical quality (polyphenols, caffeine, theanine, chlorophyll) and improve Matcha color → raise field yield, with PGPR + fertilizer increasing production by 15.38% and PGPR alone increasing yield by 92.31% compared with no input.	[81]
*Bacillus amyloliquefaciens* GB03	Perennial ryegrass (*Lolium perenne* L.)	Significantly improved shoot fresh weight, dry weight, relative water content (RWC), and chlorophyll content under severe drought (20-day natural drought).—Decreased leaf relative electric conductivity (REC) and malondialdehyde (MDA) content.—Synergistic with WRA: Further enhanced chlorophyll content compared to GB03 or WRA alone.—After 7-day rewatering: Significantly increased plant survival rate, biomass, RWC, and maintained chlorophyll content; GB03+WRA showed superior effects on survival rate, biomass, and chlorophyll content vs. control and single treatments.	GB03 colonizes the rhizosphere → enhances root water uptake, osmotic adjustment, and chlorophyll stability → reduces membrane damage (lower REC) and oxidative stress (lower MDA) → WRA slows soil water loss and maintains moisture → GB03 + WRA synergistically preserve photosynthesis and hydration during drought → plants maintain higher biomass, RWC, and chlorophyll → after rewatering, improved physiological stability enables rapid recovery → higher survival rate, biomass, and sustained chlorophyll content.	[82]
*Pseudomonas fluorescens* (M1), *P. putida* (M2), *Bacillus subtilis* (M3)	Soybean (*Glycine max* L.) cultivars Crawford, Giza111, Clark	Increased final germination percent (up to 95% in Crawford with M1); reduced mean germination time (M1 and M2). Increased stem length and shoot fresh weight (M1 best, then M2, then M3) under 200 and 400 mM NaCl. Increased chlorophyll and soluble proteins; increased proline. Reduced salinity damage via higher antioxidant enzymes, especially in tolerant Crawford. Genetic diversity shown by SDS-PAGE and RAPD/ISSR; maximum 17 bands in Crawford.	PGPR colonize the seed surface and rhizosphere → produce auxins and gibberellins that accelerate germination and seedling elongation → enhance osmotic adjustment via increased proline → boost antioxidant enzymes, reducing ROS, REC, and MDA → maintain chlorophyll and soluble proteins under salinity → improve water balance and nutrient uptake → strengthen stress-responsive protein expression (SDS-PAGE) and genetic activation (RAPD/ISSR) → result in higher germination, faster emergence, greater biomass, and superior salt tolerance, especially in the tolerant cultivar Crawford.	[83]
*Pseudomonas fluorescens* (B1), Liquid organic fertiliser (O1)	Fenugreek (*Trigonella foenum-graecum* L.)	Interaction (B1O1) gave highest soil available nitrogen and phosphorus, plant height, branches, pods, seeds per pod, choline seed content, and biological yield.	B1O1 inoculation introduces *Pseudomonas fluorescens* and *Glomus mosseae* into the rhizosphere → bacteria solubilize P and mineralize N while AMF expand the nutrient absorption zone → soil available N and P increase → enhanced root growth and nutrient uptake → microbial phytohormones stimulate plant height, branching, and pod formation → improved N and P assimilation boosts seed development and choline content → synergistic nutrient–hormone–mycorrhiza interaction maximizes biological yield.	[84]
*Azospirillum brasilense*	Purple maize (*Zea mays* L.)	Increased plant height by 10.5%, root length by 16.7%, aboveground fresh biomass by 21.3%, root fresh biomass by 30.1%, and leaf nitrogen by 27.7% vs. non-inoculated control. Improved yield by 21.8% and cob weight by 11.6%. Inoculation with 90 kg N ha^−1^ matched or exceeded non-inoculated 120 kg N ha^−1^ in height, leaf N, and cob size. Enabled 30 kg N ha^−1^ reduction with equivalent performance.	PGPR colonize the rhizosphere → enhance nitrogen uptake and assimilation → stimulate root growth and nutrient foraging → increase chlorophyll formation and photosynthetic efficiency → boost biomass accumulation and cob development → improve nitrogen use efficiency so that 90 kg N ha^−1^ with inoculation matches or exceeds 120 kg N ha^−1^ without inoculation → enable a 30 kg N ha^−1^ fertilizer reduction with equivalent yield performance.	[85]
*Bacillus* sp.	Winter wheat (*Triticum aestivum* L.)	Under drought: highest CO_2_ assimilation, minimal transpiration decline, highest stomatal conductance. Lowest initial fluorescence, highest maximum fluorescence; reduced stress parameter decline. Increased photon use efficiency and fastest electron transport in photosystems. Highest grain yield and best drought stress resistance index among biostimulants.	Biostimulant application enhances stomatal conductance and maintains CO_2_ assimilation → stabilizes transpiration and leaf water status → protects PSII and PSI by lowering initial fluorescence and increasing maximum fluorescence → accelerates electron transport and improves photon use efficiency → preserves photosynthetic capacity under drought → sustains biomass production and grain filling → results in the highest grain yield and strongest drought stress resistance index.	[86]
*Pseudomonas* spp., *Bacillus* spp.	Peppermint (*Mentha piperita* L.)	Under moderate drought: inoculation matched effects of inoculation alone or moderate stress alone on trichome density, essential oil (EO) main components, and total EO yield. No change in volatile organic compound emissions vs. uninoculated stressed plants. Enhanced secondary metabolite yield without compromising productivity under combined stress and inoculation.	PGPR colonize the rhizosphere → improve water and nutrient uptake under moderate drought → stabilize photosynthesis and reduce oxidative stress → maintain biomass production → stimulate trichome development and enhance EO biosynthetic pathways → preserve EO composition and yield without altering VOC emissions → enable higher secondary metabolite output without reducing plant productivity under combined drought and inoculation.	[87]
*Azospirillum brasilense*	Sugarcane (*Saccharum* spp.) cv. Mex 69–290 (micropropagated)	Dose-dependent effects during ex vitro acclimatization (60 days). Optimal at 1 × 10^6^ CFU/mL: high survival, enhanced growth, dry matter, chlorophyll, β-carotene; increased N, P, Mg, Mn, B uptake. Higher dose (2 × 10^6^ CFU/mL) reduced survival and development. Improved plantlet quality for field transplant.	PGPR colonize the root zone at an optimal density → enhance nutrient solubilization and uptake (N, P, Mg, Mn, B) → stimulate root and shoot development through balanced phytohormone production → increase chlorophyll and β-carotene, improving photosynthetic efficiency → strengthen stress tolerance during ex vitro transition → produce vigorous, high-quality plantlets suitable for field establishment; excessive inoculum disrupts this balance and reduces survival.	[88]

### 3.2. Ammonification

Ammonification, a critical phase in nitrogen mineralization, involves the microbial breakdown of organic nitrogen compounds from plant and animal residues into NH_3_ or NH_4^+^_. This process replenishes the soil inorganic nitrogen pool, making nitrogen available for plant uptake or for further microbial transformations.

The transformation of organic nitrogen in soil occurs through mineralization, comprising proteolysis and ammonification. Mineralization is essential for sustainable agriculture, as it enhances crop residue management, reduces dependency on synthetic fertilisers, and minimizes groundwater contamination risks [89]. Narrow C:N ratios in organic matter accelerate mineralization, increasing nitrogen availability for plants.

Proteolysis, the initial step in mineralization, involves the enzymatic breakdown of proteins into peptides and amino acids. Proteolytic enzymes are produced by soil microorganisms, including bacteria (e.g., *Bacillus*, *Pseudomonas*) and fungi (e.g., *Aspergillus*, *Penicillium*), with their activity influenced by soil pH, moisture, temperature, and organic matter availability [90]. Key enzymes, such as alkaline serine endopeptidases, subtilisin-like proteinases, and metalloproteinases, exhibit varying activity based on environmental conditions. Protease activity is greatest in nutrient-rich soils and diminishes with increasing depth, salinity, and soil compaction [91].

Ammonification follows proteolysis, wherein low-molecular-weight organic nitrogen compounds like amino acids and urea are further decomposed to NH_3_/NH_4^+^_. This process is driven by deaminases (e.g., asparaginase, urease) and amino acid oxidases secreted by microorganisms [92,93]. Ammonium ions formed can be absorbed by plants or microorganisms, retained in the soil solution, or adsorbed onto soil particles. The ammonification process is influenced by environmental factors, including temperature, pH (optimal at 6.5–8.5), and soil conditions. It is facilitated by a diverse microbial community, including bacteria (e.g., *Clostridium*, *Serratia*), actinobacteria (e.g., *Streptomyces*), and fungi (e.g., *Trichoderma*) [14]. This intricate interplay of microbial processes ensures the recycling of organic nitrogen, supporting plant nutrition and soil fertility while promoting sustainable agricultural practices.

### 3.3. Nitrification

Nitrification is a critical aerobic process in the nitrogen cycle that transforms NH_4^+^_ into NO_3^−^_. This two-step microbial oxidation plays a pivotal role in soil fertility, plant nutrition, and the dynamics of nitrogen in ecosystems [94]. However, it also contributes to nitrate leaching, a process with environmental consequences, such as groundwater contamination and eutrophication.

#### 3.3.1. Ammonium Oxidation

The first step involves the conversion of NH_4^+^_ to nitrite (NO_2^−^_). This reaction is mediated by ammonia-oxidizing bacteria (AOB) and ammonia-oxidizing archaea (AOA). Key microorganisms include species of *Nitrosomonas* and *Nitrosospira*. The oxidation of NH_4^+^_ is catalyzed in a 2-step reaction by ammonia monooxygenase (AMO) and hydroxylamine oxidoreductase (HAO) [95]:

Step 1: NH_4^+^_ + O_2_ + 2H^+^ + 2e^−^ → NH_2_OH + H_2_O

Step 2: NH_2_OH + H_2_O → NO_2^−^_ + 4H^+^ + 4e^−^

#### 3.3.2. Nitrite Oxidation

Nitrite is further oxidized to NO_3^−^_ by nitrite-oxidizing bacteria. The most studied nitrite-oxidizing bacteria are species of *Nitrobacter* and *Nitrospira*. This reaction is catalyzed by the enzyme nitrite oxidoreductase, according to the following reaction [96]:

NO_2^−^_ + H_2_O → NO_3^−^_ + 2H^+^ + 2e^−^

Nitrification rates are influenced by several key factors that collectively determine the efficiency of this aerobic process. Oxygen availability is critical, as both steps of nitrification depend on adequate oxygen levels. Soil pH also plays a significant role, with optimal activity occurring in neutral to slightly alkaline conditions, typically within a pH range of 6.5 to 8. Temperature further affects the process, as the activity of AOB and NOB increases with rising temperatures, reaching peak performance between 25 °C and 35 °C [97,98]. Additionally, sufficient NH_4^+^_ availability is essential to initiate and sustain the nitrification process, ensuring that the necessary substrate is present for these reactions to occur [99].

### 3.4. Denitrification

Denitrification is a microbially mediated process in which NO_3^−^_ is reduced, ultimately producing molecular nitrogen through a sequence of intermediate gaseous NO compounds. Facultative anaerobic bacteria carry out denitrification as a form of respiration, where oxidized nitrogen compounds serve as electron acceptors while organic matter acts as the electron donor. The sequence of nitrogen electron acceptors, ordered by thermodynamic favourability, includes NO_3^−^_, NO_2^−^_, NO, and N_2_O, culminating in the formation of molecular nitrogen, thereby completing the nitrogen cycle. This process requires very low oxygen concentrations (<10%) and an organic carbon source for energy. Given that denitrification decreases NO_3^−^_ levels, thus potentially minimizing its leaching into groundwater, it is strategically employed in treating nitrogen-rich waste, such as sewage or animal residues. However, denitrification can emit N_2_O, a greenhouse gas and ozone-depleting substance, significantly contributing to global warming [100].

Primarily, heterotrophic bacteria such as *Paracoccus denitrificans* and various species of *Pseudomonas* perform denitrification, although autotrophic denitrifiers, like *Thiobacillus denitrificans*, have also been identified. These denitrifying microorganisms are found across all major phylogenetic groups [101]. Typically, multiple bacterial species contribute to the complete reduction of NO_3^−^_ to N_2_, utilizing more than one enzymatic pathway.

An alternative pathway for NO_3^−^_ reduction involves direct conversion to NH_4^+^_, known as dissimilatory nitrate reduction to ammonium (DNRA), a process that is less prevalent than denitrification in most ecosystems. Several genes coding for nitrite reductase (such as nrf and nir) and nitrous oxide reductase (nos) are associated with denitrification. Microorganisms containing these genes include *Alcaligenes faecalis*, *Alcaligenes xylosoxidans*, multiple species of the genus *Pseudomonas*, *Bradyrhizobium japonicum*, and *Blastobacter denitrificans* [102].

### 3.5. Anammox (Anaerobic Ammonium Oxidation)

Anammox is a process where specialized bacteria (related to *Planctomycetes*) oxidize NH_4^+^_ using NO_2^−^_ as the electron acceptor, producing nitrogen gas. This anaerobic process contributes to nitrogen loss from soils and aquatic systems, particularly in oxygen-limited environments. Initially, anammox bacteria were discovered in artificial environments, but subsequent research revealed their widespread presence across diverse natural ecosystems, including anoxic marine and freshwater sediments, water columns, terrestrial environments, and unique settings such as petroleum reservoirs [103].

Studies estimate that anammox contributes to approximately 50% of nitrogen loss in marine systems. More recently, it has been reported that anammox accounts for 9–40% of nitrogen removal in inland lakes and 4–37% in agricultural soils, highlighting its significant role in freshwater and terrestrial environments. The distribution and activity of different anammox bacteria vary across ecosystems, strongly influenced by local environmental conditions [103].

Industrial application faces challenges, primarily due to the slow growth rate of anammox bacteria. Laboratory-scale systems overcame this limitation using sequencing batch reactors for enhanced biomass retention [104]. At an industrial level, the first full-scale anammox reactor was implemented in 2002 at the sludge treatment plant in Sluisjesdijk, Rotterdam, Netherlands [105]. This system, combined with the SHARON reactor, produced an optimal 50:50 mixture of NH_4^+^_ and NO_2^−^_ for anammox bacteria [104]. Later, the first full-scale CANON system, integrating partial nitritation (biological ammonia-to-nitrite conversion) and anammox in a single reactor under oxygen-limited conditions, was launched in Strass, Austria [106].

Currently, autotrophic nitrogen removal using anammox is applied in around 40 full-scale facilities treating ammonium-rich wastewater from industries such as food processing, tannery, semiconductor, distillery, and pharmaceuticals [107]. These systems handle varying nitrogen loads, with some treating up to 11 tons of N per day (Tongliao Meihua Industry, China; unpublished data). Promising results have also been reported for technical-scale applications treating black water digestate, manure, urine, and pharmaceutical wastewater [108].

The implementation of anammox technology requires careful design tailored to specific wastewater characteristics, as factors such as pH, temperature, salinity, organic carbon content, NO_2^−^_ and NH_4^+^_ concentrations, heavy metals, and antibiotics significantly influence process stability [109]. Future research aims to optimize process efficiency, minimize N_2_O emissions, and expand its application to diverse wastewater treatment scenarios.

### 3.6. Physicochemical Transformations

In addition to biological processes, physicochemical transformations influence nitrogen cycling. Among these transformations in nitrogen cycling are volatilization, leaching, and adsorption–desorption processes. These transformations significantly impact soil nitrogen availability and contribute to environmental concerns such as air and water pollution [110]. Understanding these processes is crucial for developing sustainable nutrient management strategies that optimize plant productivity while mitigating negative environmental effects.

#### 3.6.1. Volatilization of Ammonia

Ammonia volatilization is a major pathway of nitrogen loss from agricultural soils, particularly under high pH conditions. This process occurs when NH_4^+^_, derived from fertilisers, organic matter decomposition, or manure application, is converted into NH_3_ gas and lost to the atmosphere. The rate of volatilization is influenced by several factors, including soil pH, temperature, moisture, and the presence of urease enzymes. Alkaline soils, dry conditions, and high temperatures accelerate NH_3_ volatilization, leading to reduced nitrogen availability for plants and contributing to atmospheric pollution [111]. Ammonia volatilization can have significant environmental impacts, including the formation of secondary particulate matter in the atmosphere and nitrogen deposition in non-target ecosystems [112]. Management practices to reduce volatilization include incorporating urea-based fertilisers into the soil, using urease inhibitors, and applying fertilisers under conditions that minimize nitrogen loss [113].

#### 3.6.2. Adsorption and Desorption of Ammonium

The adsorption and desorption of NH_4^+^_, along with ammonium fixation, are critical for nitrogen retention and availability in soils. Ammonium can be adsorbed onto negatively charged clay particles and organic matter, reducing its immediate availability for plant uptake. Additionally, NH_4^+^_ can undergo fixation within the crystal lattice of certain clay minerals, such as vermiculite or illite, where it becomes trapped and less accessible for plant or microbial use [114]. The extent of NH_4^+^_ adsorption and fixation is influenced by soil properties such as CEC, pH, and organic matter content [115]. Desorption, the release of NH_4^+^_ from soil particles or clay lattices into the soil solution, occurs under conditions that favor increased microbial activity and plant uptake. However, if NH_4^+^_ is not promptly assimilated, it can undergo nitrification, leading to NO_3^−^_ leaching or NH_3_ volatilization losses [116]. Soil amendments such as biochar and zeolites have been investigated for their potential to enhance NH_4^+^_ adsorption and improve nitrogen uptake efficiency in agricultural systems [117].

#### 3.6.3. Nitrate Leaching

Leaching is another critical nitrogen transformation process, whereby NO_3^−^_, a highly mobile form of nitrogen, is transported below the root zone by percolating water. Nitrate leaching is particularly prevalent in sandy soils, where excess rainfall or irrigation facilitates its movement into groundwater [118]. Excessive NO_3^−^_ leaching poses significant environmental risks, including eutrophication of groundwater and surface waters. High NO_3^−^_ concentrations in drinking water can lead to health issues such as methemoglobinemia in infants [119]. Moreover, NO_3^−^_ runoff into aquatic ecosystems stimulates excessive algal growth, depleting oxygen levels and leading to hypoxic conditions detrimental to aquatic life [120]. Mitigation strategies for NO_3^−^_ leaching include adopting precision fertilization and irrigation techniques, using slow-release nitrogen fertilisers, and implementing cover cropping and buffer strips to enhance nitrogen retention in soils [121].

## 4. Interactions with Other Nutrients

The availability and utilisation of nitrogen in plants are closely interlinked with other essential nutrients, including phosphorus, potassium, and micronutrients such as copper. These interactions can significantly influence plant growth, nutrient uptake efficiency, and overall crop performance. Understanding these relationships is critical for optimizing fertilization strategies and improving nutrient use efficiency, particularly in sustainable agricultural practices [122].

**Figure 3:**
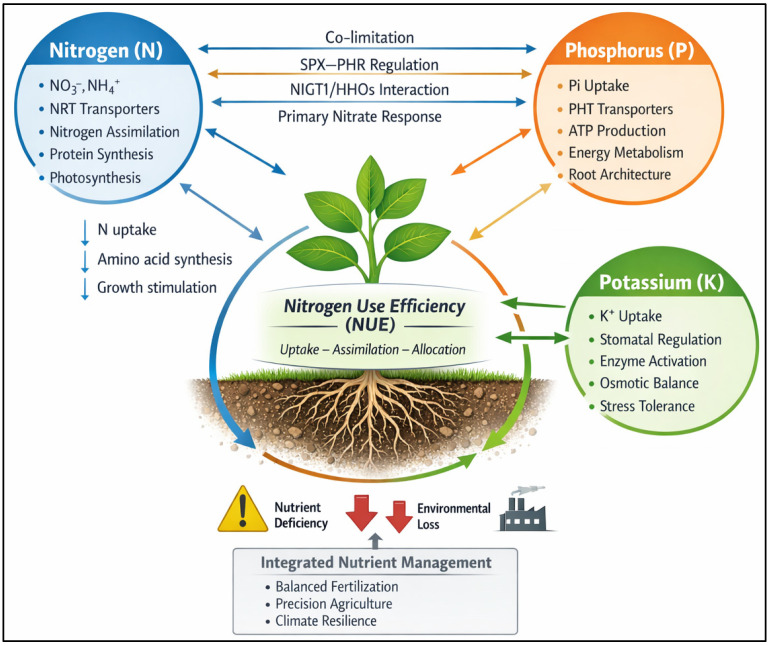
Conceptual overview of nitrogen–phosphorus–potassium (N–P–K) interactions regulating plant nutrition and nitrogen use efficiency.

Given the complexity of nitrogen–phosphorus–potassium (N–P–K) interactions at molecular, physiological, and agronomic scales, a conceptual synthesis is provided in Figure 3. The diagram distills the key synergistic and antagonistic relationships described in this section, illustrating how phosphorus availability regulates the energetic costs of nitrogen assimilation, how potassium modulates enzyme activation, osmotic balance, and nitrogen transport, and how imbalances in any one nutrient constrain the efficiency of the others. By integrating uptake, metabolism, and source–sink regulation into a single framework, Figure 3 facilitates interpretation of the mechanistic pathways underlying nutrient crosstalk and highlights leverage points for integrated nutrient management strategies aimed at improving overall nitrogen use efficiency.

### 4.1. Nitrogen and Phosphorus Interactions

Nitrogen and phosphorus interact in complex ways within soil and plant systems. High nitrogen levels can increase phosphorus demand, particularly in fast-growing crops, as nitrogen promotes vegetative growth. Excessive nitrogen fertilization may reduce phosphorus uptake by limiting root development or disrupting nutrient balance [123]. Conversely, an adequate phosphorus supply can mitigate the negative effects of excess nitrogen, improving nitrogen utilisation efficiency [124]. Excessive nitrogen application can alter soil pH, which affects phosphorus availability by promoting its fixation in soils, particularly in soils with low phosphorus buffering capacity. This increased fixation, often due to phosphorus binding with iron, aluminum, or calcium ions, reduces phosphorus availability for plant uptake [125].

In terrestrial ecosystems, nitrogen and phosphorus often limit plant productivity and food security. The Liebig’s law of the minimum suggests growth depends on the scarcest nutrient, but research highlights interactive nitrogen–phosphorus co-limitation. Meta-analyses show that combined nitrogen and phosphorus application boosts yields in crops like wheat, rice, maize, and cotton. Physiologically, nitrogen enhances phosphorus uptake, and phosphorus deficiency impairs NO_3^−^_ absorption and assimilation. Despite these insights, the molecular mechanisms underlying these interactions remain underexplored [126].

Phosphorus and nitrogen signalling pathways respond to the availability and depletion of these nutrients. Under phosphorus deficiency, the phosphate starvation response activates regulatory elements, including the non-coding RNA Induced by Phosphate Starvation 1 (IPS1), microRNA-399 (miR399), and phosphate transporter genes (PHTs). These are modulated by inositol polyphosphates (InsP) and interactions between Phosphate Starvation Response (PHR) proteins and SPX-domain proteins [127]. Nitrogen signalling includes the primary nitrate response (PNR), triggered by NO_3^−^_ resupply, with markers such as NIA1, NIR, and HRS1 [128]. Nitrate sensing involves CHL1/NRT1.1, which activates calcium-dependent pathways via CPKs [128]. The nitrogen starvation response (NSR) induces genes like NRT2.4, NRT2.5, and GDH3, controlled by NIGT1/HHOs, LBD37-39, and NFYA [129,130]. These pathways interact, with NIGT1/HHOs regulating both primary nitrate response and nitrogen starvation response, facilitating systemic nutrient signalling [131].

Nutrient availability, particularly of phosphorus and nitrogen, intricately shapes root architecture to optimize nutrient acquisition. Phosphorus deficiency enhances lateral root growth through pathways involving Low Phosphate Root 1/2 (LPR1/2) and Sensitive to Proton Rhizotoxicity 1 (STOP1), which regulate root responses to low phosphorus conditions [132]. In contrast, NO_3^−^_ availability differentially affects lateral root elongation depending on its concentration: low NO_3^−^_ levels often suppress elongation, moderate levels promote it, while high levels can inhibit it due to feedback regulation [133]. These responses are mediated by transcription factors such as Hypersensitivity to Low Phosphate-Elicited Primary Root Shortening 1 (HRS1), which modulates phosphorus-related gene expression, with mutants like arabidopsis hrs1, hho1 showing altered root architecture under combined phosphorus and nitrate limitations [134]. Similarly, nitrate transporter mutants, such as NPF7.3/NRT1.5, exhibit distinct root phenotypes under phosphorus deficiency, highlighting the interplay between nitrate and phosphorus signaling [135]. These findings, synthesized from multiple studies, underscore the complex, concentration-dependent interactions between nitrogen and phosphorus in shaping root development for efficient nutrient uptake.

Nitrogen availability regulates phosphate starvation response, with nitrogen supplementation activating it and nitrogen starvation suppressing it [136], key regulators include SPX1-4, PHRs, and PHO2. In arabidopsis, SPX1-4 are repressed by NIGT1/HHOs under nitrogen starvation [137], whereas in rice, OsSPX4 undergoes nitrate-dependent degradation via OsNBIP1 [137]. The PHR1-LIKE 1 and OsPHR3 are induced by nitrogen, but PHR1 stability decreases under nitrogen starvation [138]. The PHO2 mutants in arabidopsis disrupt nitrogen-mediated phosphate starvation response repression, indicating a role in nutrient coordination [139].

Phosphorus deficiency inhibits nitrogen uptake and assimilation. Transcriptome studies show downregulation of nitrogen-related genes (NIA1, NIR, CHL1/NRT1.1) within 24 h of phosphorus starvation [140]. Direct regulation occurs through CHL1/NRT1.1 stability and SPX4-mediated repression of OsNLP3 in rice. The OsSPX4 degradation, influenced by InsP-facilitated OsPHR interactions, ensures dynamic nitrogen–phosphorus coordination [141]. Although nitrogen–phosphorus interactions evolved in natural ecosystems, decoupling them (e.g., through pho2 or NIGT1/HHOs mutants) could enhance nutrient use efficiency (NUE, PUE) in agriculture [130,138]. Further research into these regulatory networks is essential for sustainable crop nutrition.

### 4.2. Nitrogen and Potassium

The interaction between nitrogen and potassium is typically synergistic but can be disrupted by nutrient imbalances. Sufficient potassium enhances nitrogen uptake and assimilation, improving nutrient use efficiency. By regulating stomatal opening and water movement, potassium supports nutrient absorption and minimizes nitrogen loss through leaching [142].

Potassium improves crop quality and reduces environmental pollution from nitrogen and phosphorus losses. It enhances the efficiency of nitrogen and phosphorus fertilisers, decreasing nutrient runoff [143]. Nutrient imbalances are evident in regions like China, where the nitrogen–phosphorus–potassium ratio averages 1:0.43:0.17, notably lower than the global average of 1:0.47:0.37 or the 1:0.57:0.55 ratio observed in developed countries [144].

Nitrogen and potassium differ in their chemical forms and uptake mechanisms. Plants absorb potassium exclusively as K^+^, whereas nitrogen is taken up as NH_4^+^_ or NO_3^−^_. As monovalent cations with similar ionic radii, NH_4^+^_ and K^+^ compete for soil exchange sites, potentially displacing K^+^ into the soil solution when NH_4^+^_-based fertilisers are applied, which can increase potassium availability in the short term [145,146]. However, in sandy soils with high water content, this desorbed K^+^ may be leached below the root zone, reducing its availability to plants [145].

Soil potassium availability is influenced by soil type, crop management, and fertiliser practices. Non-exchangeable potassium, trapped within the crystal lattices of clay minerals such as illite or vermiculite, serves as a long-term reservoir but is released slowly under field conditions compared to controlled experiments, limiting its immediate availability to plants [147]. Excessive reliance on nitrogen fertilisers without adequate potassium inputs can deplete exchangeable potassium pools, particularly when crop residues are removed, exacerbating deficiencies. Balanced potassium fertilization can replenish soil levels, though the replenishment of both exchangeable and non-exchangeable potassium pools occurs gradually [147].

Potassium mitigates stress from nitrogen imbalances, drought, and biotic factors. It enhances nitrogen metabolism under stress, improving plant performance [148]. Mechanistically, potassium strengthens plant defenses by limiting pathogen access to nutrients, reinforcing cell walls, and regulating stomatal function to reduce pathogen invasion [149]. It promotes the synthesis of high-molecular-weight compounds (e.g., proteins, starches) while reducing low-molecular-weight compounds linked to disease susceptibility [150]. Moreover, adequate potassium increases phenolic compound accumulation and enhances pest resistance by reducing pest attraction and increasing their mortality [151].

Drought stress limits crop productivity by restricting root growth and K^+^ mobility [152]. Insufficient potassium exacerbates drought sensitivity by impairing stomatal regulation, membrane stability, and osmotic balance. Adequate potassium improves drought tolerance through: (A) enhanced root elongation and water uptake via deep potassium placement [153], (B) optimized stomatal function and photosynthesis under water scarcity [150], and (C) improved membrane stability to mitigate dehydration damage [154].

The nitrogen–potassium interaction is critical for stress resilience. Adequate nitrogen boosts potassium uptake, promoting root development, water use efficiency, and metabolic activity under stress. In turn, potassium regulates nitrogen metabolism by enhancing protein synthesis, enzyme activity, and photosynthetic efficiency, and improving resistance to biotic and abiotic stresses [14].

Potassium–nitrogen synergy mitigates various environmental and physiological stresses, including cold, waterlogging, and excessive nitrogen application. Under cold stress, potassium enhances antioxidant activity, reduces reactive oxygen species (ROS), and improves membrane stability, thereby minimizing frost damage [148]. In rice, a higher K/N ratio reduces spikelet sterility under cold stress by enhancing membrane permeability and phospholipid content [146]. During waterlogging, potassium maintains ion homeostasis, counters membrane depolarization, and mitigates oxidative stress caused by toxic compounds, while exogenous potassium promotes growth, photosynthesis, and nutrient uptake (K^+^, Ca^2+^, nitrogen) under hypoxic conditions [146]. Excessive nitrogen application can induce physiological stress by disrupting nutrient balance, promoting excessive vegetative growth, and increasing susceptibility to lodging and diseases. Potassium counteracts these effects by optimizing protein synthesis, enhancing enzyme activity, and improving stomatal function, thus alleviating nitrogen-induced stress. This synergy also supports tolerance to drought and pathogen attacks, enhancing crop resilience, productivity, and sustainability [148].

### 4.3. Nitrogen and Micronutrients (e.g., Copper, Iron, Zinc, etc.)

Nitrogen availability influences the uptake of micronutrients such as Cu, Fe, and Zn. Nitrogen fertilization alters soil pH, organic matter, and microbial activity, which affect the solubility and mobility of Cu, Fe, and Zn in the rhizosphere [155]. Specifically, NH_4^+^_ fertilization can compete with cationic micronutrients like Cu^2+^, Fe^2+^/Fe^3+^, and Zn^2+^ for uptake pathways, such as root transporters, potentially reducing their assimilation. In contrast, NO_3^−^_ does not directly compete with these micronutrients due to its anionic nature but may indirectly influence their availability by altering soil pH and rhizosphere dynamics [14]. Understanding these interactions is vital for optimizing nutrient management to enhance crop productivity and nutrient use efficiency in sustainable agriculture.

Copper is an essential micronutrient integral to nitrogen metabolism, serving as a cofactor for enzymes like polyphenol oxidase, cytochrome c oxidase, and nitrite reductase, which support nitrogen fixation and assimilation [156]. Copper deficiency impairs these enzymatic functions, disrupting nitrogen metabolism. For example, in legumes, Cu deficiency reduces nitrogenase activity in root nodules, lowering nitrogen fixation efficiency [157]. It also inhibits nitrate reductase, limiting the conversion of nitrate to ammonium [158].

Nitrogen availability, especially in the form of NH_4^+^_, influences Cu dynamics in soil. High NH_4^+^_ uptake decreases rhizosphere pH via proton exchange, reducing Cu uptake in acidic soils (pH < 5.5), but increasing Cu solubility in neutral to alkaline soils (pH > 6.5) [159]. By contrast, NO_3^−^_-based fertilization raises soil pH via plant uptake of NO_3^−^_, which triggers OH^−^/HCO_3^−^_ release into the rhizosphere [150,160,161]. In very acidic soils (pH < 5.5), Cu is often bound tightly to organic matter or precipitated as insoluble compounds [162]. A moderate pH increase disrupts these bonds, enhancing Cu solubility and availability despite higher pH [163]. This effect depends on initial soil pH, organic matter, Cu forms, and plant root exudates [164]. Thus, balanced fertilization strategies are crucial to prevent Cu deficiencies and maintain crop productivity and quality in nutrient-limited systems.

Iron availability is limited in high-pH or calcareous soils, where it exists as insoluble Fe^3+^ [165]. Adequate NH_4^+^_ nitrogen promotes root growth and rhizosphere acidification, increasing Fe solubility and uptake. However, excessive NO_3^−^_ fertilization can raise rhizosphere pH, exacerbating Fe chlorosis in crops like soybean, maize, and citrus [166]. Nitrogen deficiency reduces Fe transporter gene expression, whereas sufficient N supply enhances Fe uptake and translocation [167]. Iron deficiency, in turn, impairs nitrate reductase activity, creating a feedback loop affecting both nutrients.

Zinc activates glutamine synthetase and nitrate reductase, facilitating nitrogen assimilation [168]. While NH_4^+^_ fertilization generally acidifies the rhizosphere, enhancing Zn solubility locally, its common co-application with phosphate fertilisers in alkaline conditions often leads to net Zn immobilization through precipitation and fixation, leading to deficiencies [169]. Nitrogen deficiency, conversely, reduces Zn uptake by limiting root activity. Zinc deficiency inhibits Zn-dependent enzymes (primarily glutamine synthetase), causing NH_4^+^_ accumulation, which can be toxic [170]. Glutamine synthetase serves as the key enzyme catalyzing the initial and rate-limiting step in NH_4^+^_ assimilation in plants, facilitating the ATP-dependent synthesis of glutamine from NH_4^+^_ and glutamate [171]. Inhibition of glutamine synthetase activity resulting from Zn deficiency disrupts this primary pathway for NH_4^+^_ detoxification and incorporation. Consequently, even when NH_4^+^_ is absorbed from the soil or produced endogenously, its efficient conversion into amino acids is impaired in the absence of functional glutamine synthetase. This leads to a severe metabolic impasse, resulting in the buildup of free NH_4^+^_ to cytotoxic concentrations within the cytosol [172].

## 5. Consequences of Nitrogen Mismanagement

Nitrogen fertilization has been fundamental to increasing global agricultural productivity; however, persistent overapplication has disrupted natural nitrogen cycling, generating cascading environmental and human health consequences. Excess reactive nitrogen, including NO_3^−^_, NH_3_, NO_x_, and N_2_O, escapes agroecosystems through leaching, runoff, and gaseous emissions. These losses drive soil degradation, water and air pollution, greenhouse gas emissions, biodiversity loss, and direct health risks, underscoring the urgency of integrated and sustainable nitrogen management strategies [173,174].

### 5.1. Mechanisms of Nitrogen Toxicity and Environmental Dissemination

Following application, nitrogen fertilisers undergo microbial transformations—mineralization, nitrification, and denitrification—that determine their environmental fate. Nitrate, being highly mobile, readily leaches into groundwater or is transported via surface runoff into rivers, lakes, and coastal waters. Ammonia volatilization and NO_x_ emissions contribute to atmospheric nitrogen deposition and secondary particulate formation, while denitrification produces N_2_O, a potent greenhouse gas with a global warming potential approximately 273 times that of CO_2_ over a 100-year horizon [175]. In humans, ingested nitrate is reduced to nitrite (NO_2^−^_), which oxidizes hemoglobin to methemoglobin, impairing oxygen transport. Infants under six months are particularly vulnerable due to immature enzymatic detoxification systems, leading to infant methemoglobinemia (“blue-baby syndrome”) [176]. Chronic exposure has also been associated with increased risks of certain cancers and cardiovascular disorders [177].

### 5.2. Aquatic and Terrestrial Ecosystem Impacts

Excess nitrogen loading has profoundly altered both aquatic and terrestrial ecosystems. In freshwater and coastal systems, nitrate-rich runoff stimulates eutrophication, triggering harmful algal blooms, hypoxia, and biodiversity loss [178,179]. Harmful cyanobacterial blooms frequently release potent toxins, including microcystins and saxitoxins, while simultaneously elevating nitrate and nitrite concentrations. These changes threaten aquatic ecosystems and may endanger human health through exposure to contaminated drinking water and aquatic food products [180]. In terrestrial ecosystems, chronic nitrogen enrichment leads to soil acidification, nutrient imbalances, and altered microbial functioning. Forests and grasslands receiving elevated nitrogen inputs often experience reduced plant species richness, as nitrogen-tolerant species outcompete slower-growing taxa, weakening ecosystem resilience [181,182]. Microbial processes regulating nitrogen turnover are strongly modulated by climate drivers. Warming and altered precipitation regimes influence mineralization, nitrification, and denitrification rates, increasing uncertainty in nitrogen loss pathways via leaching and gaseous emissions [183,184].

### 5.3. Greenhouse Gas Emissions and Climate Feedback

Anthropogenic nitrogen inputs have more than tripled global reactive nitrogen creation since pre-industrial times, intensifying nitrogen-driven greenhouse gas emissions across ecosystems [185,186]. Agricultural soils account for approximately 60% of global anthropogenic N_2_O emissions, with emissions increasing exponentially as fertiliser rates rise [175,187]. While nitrogen deposition may temporarily enhance plant productivity and carbon sequestration, these benefits are frequently offset by increased N_2_O emissions and reduced methane oxidation, resulting in net warming feedbacks over time [188,189]. Long-term experiments indicate that nitrogen saturation and microbial acclimation can reverse initial carbon sinks into sources, complicating climate mitigation strategies [190].

### 5.4. Soil Degradation, Salinity, and Biodiversity Loss

Excessive nitrogen fertilization accelerates soil degradation through acidification, nutrient leaching, and salinization. Ammonium-based fertilisers increase proton release during nitrification, lowering soil pH and mobilizing toxic metals that impair microbial activity and root function [191,192]. Nitrogen-induced salinity arises from the accumulation of soluble salts such as nitrates and sulfates, particularly under inefficient irrigation and poor drainage. Salinity disrupts nitrogen uptake and assimilation by inhibiting nitrate reductase and glutamine synthetase, reducing protein synthesis and crop yields [14]. Soil biodiversity declines under intensive nitrogen inputs, compromising ecosystem services such as carbon storage, water retention, and nutrient cycling. Sustainable practices—including crop rotation, reduced tillage, and agroforestry—have been shown to restore soil biological integrity and mitigate nitrogen-driven degradation [193,194].

### 5.5. Human Health and Air Quality Implications

Mismanagement of nitrogen in agricultural systems poses significant and multifaceted risks to human health through contaminated drinking water, degraded air quality, and indirect effects mediated by food systems and ecosystems. One of the most direct exposure pathways is NO_3^−^_ leaching into groundwater, particularly in intensively fertilized regions. Elevated nitrate concentrations in drinking water are well established as a cause of infant methemoglobinemia [176]. To mitigate this risk, regulatory agencies such as the U.S. Environmental Protection Agency have established a maximum contaminant level of 10 mg L^−1^ NO_3^−^_ in drinking water [195]. Beyond acute toxicity, chronic NO_3^−^_ exposure has been increasingly linked to long-term health outcomes. Epidemiological studies associate sustained ingestion of NO_3^−^_-contaminated water with increased risks of colorectal, gastric, bladder, and thyroid cancers, largely due to endogenous formation of N-nitroso compounds, which are recognized carcinogens [177]. Additional associations have been reported with adverse pregnancy outcomes, including preterm birth and low birth weight, as well as thyroid dysfunction and hypertension in adults [177].

Nitrogen mismanagement also substantially degrades air quality through emissions of NH_3_ from fertilized soils and livestock systems. Atmospheric NH_3_ reacts with nitrogen oxides and sulfur dioxide to form fine particulate matter (PM_2.5_), a major contributor to global disease burden. PM_2.5_ penetrates deep into the lungs and bloodstream, triggering systemic inflammation, oxidative stress, and endothelial dysfunction, thereby increasing the incidence of asthma, chronic obstructive pulmonary disease, ischemic heart disease, stroke, and lung cancer [196,197]. Regions with intensive nitrogen fertiliser use consistently show elevated PM_2.5_ concentrations and associated premature mortality rates [198]. Harmful algal blooms stimulated by excess nitrogen produce cyanotoxins such as microcystins, cylindrospermopsin, and saxitoxins, which contaminate drinking water supplies and aquatic food products. Exposure to these toxins has been linked to acute liver damage, neurotoxicity, gastrointestinal illness, and increased long-term cancer risk [179,180]. Furthermore, nitrogen accumulation in crops, particularly leafy vegetables, can elevate dietary NO_3^−^_ intake. While NO_3^−^_ from vegetables can have cardiovascular benefits under controlled intake, excessive accumulation—often driven by high fertiliser rates and poor nitrogen timing—raises concerns when combined with other exposure sources, especially in vulnerable populations [199].

## 6. Cutting-Edge Strategies for Enhancing NUE and Mitigating Environmental Impact

**Figure 4:**
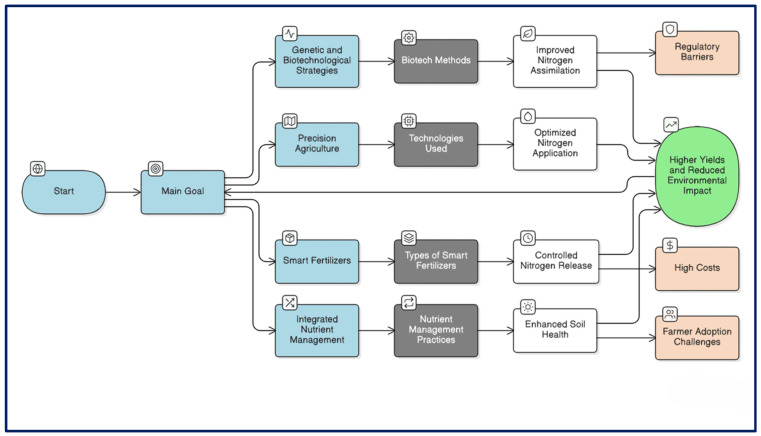
Flowchart of the circular nitrogen economy, showing recycling strategies (e.g., composting, wastewater recovery), their benefits (e.g., reduced emissions, economic savings), and challenges (e.g., technological barriers), emphasizing a closed-loop system.

Modern agriculture faces the dual challenge of feeding a growing global population, projected to reach 10 billion by 2050, while addressing climate change and soil degradation [200,201]. With additional 2 billion people to feed, it is unsustainable to consider expanding farmland or increasing nutrient inputs [202]. Innovative strategies (including precision agriculture, integrated nutrient management, and smart fertilisers) (Figure 4) and interdisciplinary colaborateion are essential to enhance productivity on existing land while reducing nitrogen losses and environmental impacts [203].

### 6.1. Precision Agriculture Technologies

Precision agriculture (PA) leverages technologies like GPS, sensors, Geographic Information Systems (GIS), and precision equipment (Table 2) to optimize nitrogen management, improving nitrogen use efficiency, productivity, and environmental sustainability [204]. Emerging in the 1980s, PA tailors nitrogen applications to site-specific field conditions, addressing soil and crop variability [205]. High-resolution (<10 m) data from satellite imagery, drones, and soil sensors define management zones, while advanced computing enables real-time decision-making [206]. Site-specific fertiliser adjustments boost nitrogen use efficiency by up to 368% compared to uniform applications, reducing losses from leaching, volatilization, and denitrification [207]. GPS-guided autosteer systems improve application efficiency by 5–10% by minimizing overlaps, and integrating sensors with GIS for variable-rate nitrogen application saves 10–20% on inputs, depending on field variability [208]. However, challenges include high initial costs, data management needs, and requirements for technical expertise [204]. Future advancements in artificial intelligence (AI), machine learning, and Internet of Things (IoT) are expected to enable real-time nitrogen adjustments based on dynamic field conditions [209].

The PA also enhances water use efficiency through variable rate irrigation (VRI), which applies water based on soil and crop needs, reducing drainage and nutrient leaching [210]. The VRI uses electromagnetic soil mapping and wireless sensor networks to create irrigation prescription maps, accounting for soil texture, moisture variability, and real-time data [211]. Modelling shows water savings of 9–26%, improved energy efficiency, and drainage reductions of 0–55%, lowering nitrogen leaching risks [212]. Despite its potential, global VRI adoption is limited, requiring improved decision-support systems for management zones and adaptive irrigation control [213].

Precision fertigation, integrating nutrient delivery with irrigation, enhances efficiency by applying water and nutrients simultaneously. It enables uniform or variable application over large areas (≥50 ha), with high automation and cost-effective delivery across diverse soils and crops [214]. However, fertigation requires precise prescription maps for water and nutrient needs, as soil dryness may not indicate fertiliser requirements [212]. Even though drip irrigation is efficient, it is costly and maintenance-intensive [214]. Trials in Australia with sub-surface drip irrigation for pastures show reduced nitrate leaching and nitrous oxide emissions, though soil acidification remains a concern [213]. The VRI and precision fertigation offer promising solutions for enhanced resource-use efficiency, but further research is needed to overcome adoption barriers and refine decision-support tools, ensuring sustainable nitrogen management and enhanced agricultural productivity [215].

**Table 2:** Impacts of precision agriculture (PA) technologies on yield and product cost of various crops.

PA Technology	Crop	Impacts	References
Variable-rate fertilization	Maize	Consistently improves N use efficiency and maintains or modestly increases yield when spatial variability is high; effects are site-specific.	[121]
Variable-rate fertilization	Wheat	Small to moderate yield gains reported; strongest benefits observed in reduced fertilizer inputs rather than yield.	[121]
Variable-rate fertilization	Rice	Evidence primarily relates to reduced N losses and improved input efficiency rather than yield response.	[121]
Remote sensing/satellite imagery	Soybean	Improves in-season nutrient and stress diagnostics, supporting timely management decisions; yield effects variable.	[204]
GPS-guided equipment	Wheat	Reduces overlap and input waste; yield effects indirect via improved operational efficiency.	[216]
Soil sensors and IoT systems	Maize	Reductions in nitrate leaching and improved irrigation–fertilizer synchronization reported.	[217]
Drones and UAVs (Unmanned Aerial Vehicles)	Rice, citrus, grapes	Rice: Drones detected nitrogen deficiencies, improving yield by 15%. Citrus: Aerial imagery optimized nitrogen application, enhancing fruit quality. Grapes: Precision nitrogen management improved vine health and grape yield.	[218]
Yield monitoring systems	Wheat, soybean, maize	Wheat: Yield maps identified low-nitrogen zones, improving NUE by 20%. Soybean: Optimized nitrogen application increased yield by 10%. Maize: Reduced nitrogen over-application, saving 15% on fertiliser costs.	[219]
Automated Irrigation Systems (AIS)	Vegetables, rice, citrus	Vegetables: AIS reduced nitrogen leaching by 25%. Rice: Alternate wetting and drying (AWD) reduced water and nitrogen use by 30%. Citrus: AIS improved fruit size and quality.	[220]
Decision Support Systems (DSS)	Wheat, maize, potato	Wheat: DSS optimized nitrogen application timing, increasing yield by 15%. Maize: Reduced nitrogen losses by 20% through data-driven decisions. Potato: Improved tuber yield and quality with precise nitrogen management.	[221]

### 6.2. Genetic and Biotechnological Strategies

Genetic and biotechnological approaches, including advanced breeding, genome editing, and microbial inoculants, offer innovative solutions to improve nitrogen assimilation, fixation, and utilisation, reducing reliance on synthetic fertilisers and mitigating environmental harm (Table 3).

#### 6.2.1. Enhancing Nitrogen Assimilation Efficiency Through Crop Breeding and Genome Editing

Crop breeding has progressed significantly with genomics-assisted approaches, such as marker-assisted selection (MAS), which targets genomic regions associated with nitrogen metabolism to create varieties exhibiting enhanced nitrogen use efficiency. This enhances yields in crops like rice and wheat under low-nitrogen conditions, boosting productivity, reducing farmer costs, and promoting environmental sustainability [222,223]. However, challenges include the time-consuming process spanning multiple generations, the genetic complexity of polygenic traits, and potential trade-offs with traits like pest resistance. It scales better in temperate, large-scale farming with robust infrastructure than in tropical smallholder systems, where diverse microclimates and limited resources impede adoption [223]. Effectiveness ranges from 10 to 20% yield increases, varying by crop and region, with high initial costs but lower long-term expenses, making it viable in advanced regions like North America and China but less so in sub-Saharan Africa.

Genome editing, particularly CRISPR-Cas9, targets nitrogen pathway genes like nitrate transporters (NRT1.1, NRT2.1) and enzymes (glutamine synthetase, glutamate synthase) for precise improvements, achieving 15–30% nitrogen uptake efficiency gains in crops like rice and barley. It offers faster, more precise results than traditional breeding, reduces fertiliser use, and adapts across species [224,225]. Drawbacks include technical complexity requiring specialized facilities and public acceptance issues. It is effective in GMO-friendly, temperate large-scale systems but struggles in tropical smallholder settings due to infrastructure and regulatory constraints. Effectiveness is high in controlled environments, but field data is limited, with high initial costs offset by moderate recurring ones, feasible mainly in biotech hubs.

Microbial inoculants, using nitrogen-fixing bacteria like *Rhizobium*, improve soil nitrogen availability through symbiotic fixation, meeting 20–50% of crop needs. They are affordable, easy to apply via seed coating, reduce environmental pollution, and suit smallholders [226,227]. However, performance varies with soil and climate and inoculum storage issues in tropics and repeated applications increase labor. They are effective in tropical smallholder legume systems due to simplicity and low cost, where fertilisers outperform. Effectiveness is higher in nitrogen-poor soils, with minimal costs and high feasibility for resource-constrained farms [228]. In comparison, breeding offers moderate effectiveness and wide applicability but is slow; genome editing provides high precision but faces cost and regulatory barriers. Integrating these approaches—such as developing nitrogen use efficiency-enhanced cultivars compatible with inoculants—could optimize results across agroecosystems. Large-scale farms in temperate regions benefit most from breeding and gene editing, whereas tropical smallholders gain from inoculants [229].

#### 6.2.2. Biotechnological Tools

Biotechnological advancements like microbial inoculants and biofertilisers significantly enhance nitrogen fixation and assimilation in crops. Nitrogen-fixing bacteria convert atmospheric nitrogen into forms plants can use, thereby improving nitrogen agronomic efficiency [227,230]. PGPR produce phytohormones and enzymes that boost root growth and nutrient uptake, further aiding nitrogen assimilation [226,231].

**Table 3:** Effects of genetic and biotechnological strategies on nitrogen use efficiency (NUE) in plants.

Strategy	Crop	Effect on NUE	Targeted Genes	Findings	References
Novel Plant Breeding Techniques (NPBTs) and Genome Editing (GETs)	Rice, arabidopsis, *Medicago truncatula*	Precise improvement of NUE-related genes, accelerated crop improvement	OsNRT1.1b (OsNPF6.5), OsNRT2.3a, OsGS1;1, AtNRT1.1, and AtGS2	Genome editing tools such as CRISPR/Cas9, base editing, and prime editing have been applied in model plants to improve NUE by targeting N transporter and assimilator genes. Enhanced mutation efficiencies (up to 95%) achieved with optimized promoters like arabidopsis UBQ10 improve gene editing success rates. Base editing successfully improved NUE traits in rice, demonstrating promising routes for sustainable agriculture.	[232,233]
Marker-Assisted Selection (MAS)	Wheat	Efficient incorporation of NUE traits into breeding lines	TaNRT2.1, TaAMT1;2, TaGS1, and TaGOGAT	MAS accelerates nitrogen uptake, assimilation, and tolerance to low N conditions in wheat. High-throughput genotyping facilitate precise selection. It reduces time and cost while improving yield and NUE.	[234]
Exploration of natural genetic variability	Sorghum	Identification of high NUE genotypes adaptable to varying N regimes	SbAMT, SbNRT, and SbGS	In sorghum, significant genetic variation exists for NUE traits under low and moderate nitrogen levels. Contrasting genotypes exhibited distinct expression of N transporter and assimilatory genes (e.g., *SbAMT*, *SbNRT*, *SbGS*). Such genotypes are promising candidates for breeding N-efficient cultivars adaptable to low input systems.	[235]
Microbial inoculants (PGPB)	Maize	Increased grain yield and NUE across nitrogen rates	ZmNRT, ZmAMT, and ZmGln1	Inoculation with *Azospirillum brasilense* significantly increased maize yield, leaf chlorophyll, nitrogen content, and NUE regardless of N source; highest economic returns at 100 kg N/ha + inoculation.	[236]
Combined application of microbial inoculants with organic fertilisers	Barley	Synergistic enhancement of NUE and crop yield under suboptimal soil conditions	HvGS and HvGOGAT	Combined use of *Kosakonia radicincitans* and organic fertilisers improved barley grain yield and nutrient uptake significantly in acidic and low-P soils, highlighting integrated soil microbiome management to enhance NUE.	[237]
Microbial inoculants (PGPB & Rhizobia)	Soybean	Enhanced nodulation and symbiotic N fixation under drought	GmNOD, GmGS, and GmGOGAT	Drought-tolerant *Sinorhizobium fredii* strain improved nodule number, water potential, and reduced oxidative damage under water deficit, boosting biological nitrogen fixation efficiency.	[238]
Microbial inoculants (PGPB)	Tomato	Improved nitrogen uptake and plant growth under fertilization	SlAMT, SlNRT, and SlGS	*Bacillus pumilus* inoculation enhanced soil ammonium levels, rhizobacterial populations, *nifH* gene expression, and nitrogenase activity, leading to increased nitrogen uptake and biomass under additional N supply conditions.	[239]
Microbial inoculants (PGPB & Rhizobia)	Pea	Enhanced nodulation, biomass, and yield components	PsNOD, PsGS, and PsAAT	Application of *Rhizobium* bio-fertiliser optimized nodulation and produced the highest yield in Ethiopian highlands.	[240]
Microbial inoculants (PGPB & Rhizobia)	Pea	Significant increase in grain yield via biological nitrogen fixation	PsGS/GOGAT	Comprehensive review and meta-analysis showed rhizobia inoculation increased pea grain yield by approximately 33% compared to uninoculated controls.	[241]

### 6.3. Recent Advances in Smart Fertilisers

Smart fertilisers utilize advanced technologies like controlled-release mechanisms, nanotechnology, and polymer coatings to enhance nitrogen use efficiency, optimize plant growth, and reduce environmental impact. By addressing the inefficiencies of conventional fertilisers, these innovations minimize nutrient losses, improve crop yields, and promote sustainable agriculture.

#### 6.3.1. Controlled-Release Fertilisers

Controlled-release fertilisers (CRFs) deliver nutrients gradually, aligning with plant needs and soil conditions such as moisture, temperature, and microbial activity. Encapsulated in coatings like polymers or sulfur, CRFs regulate nutrient release through diffusion or microbial degradation, reducing losses from leaching and volatilization [242]. This enhances NUE by up to 50%, maintains or increases crop yields, and minimizes environmental impacts like NO_3^−^_ leaching and N_2_O emissions [243]. For example, polymer-coated urea increased NUE by 20–30% in maize and improved yields by 10–15% in water-limited wheat crops [244]. The CRFs also enhance crop nutritional quality, e.g., in tomatoes and maize [245].

The CRFs are particularly effective in high-rainfall areas and sandy soils, where they reduce nutrient leaching, and for perennial crops like fruit trees, ensuring consistent nutrient supply. Integration with precision agriculture, such as variable-rate systems, further optimizes nutrient delivery [246]. Despite benefits, the adoption is hindered by high production costs, regulatory concerns over non-biodegradable coatings, and limited farmer awareness [242,243]. Future research should focus on cost-effective, biodegradable coatings, long-term field studies, and improved farmer education through extension services [54,246,247].

#### 6.3.2. Nanotechnology Applications in Fertilisers

Nanofertilisers use nanoparticles to enhance nutrient delivery, improving nitrogen utilisation efficiency and reducing environmental losses. Nanofertilisers like nano-encapsulated urea synchronize nutrient release with plant demand, reducing losses by up to 50% and decreasing N_2_O emissions by 30% compared to conventional urea [139,248]. Nanoparticles such as hydroxyapatite and chitosan stabilize nitrogen, extending availability and minimizing leaching [139]. Nanofertilisers also respond to environmental triggers such as pH or enzymatic activity, optimizing nutrient release [249].

Nanofertilisers enhance crop resilience to stresses like drought and salinity. For instance, zinc oxide nanoparticles improve drought tolerance by regulating stomatal function and antioxidant activity [250], and nano-encapsulated nitrogen fertilisers enhance uptake in saline soils [245]. Despite their potential, the adoption is limited by high production costs, lack of standardized manufacturing, and concerns about nanoparticle accumulation in soil and water [245,250]. Future research should develop cost-effective, biodegradable nanomaterials and evaluate long-term environmental impacts to ensure safety and scalability [251].

#### 6.3.3. Polymer-Coated or Smart Gel Fertilisers

Polymer-coated and smart gel fertilisers use hydrogels or biodegradable polymers to release nutrients gradually, aligning with plant needs and soil moisture levels. This reduces leaching, volatilization, and runoff, improving nitrogen uptake efficiency by 20–30% and nitrate leaching by up to 40% compared to conventional fertilisers [252]. These fertilisers also enhance soil moisture retention, reducing irrigation needs in arid regions and improving yields by 10–15% in crops such as wheat [253]. Biodegradable coatings minimize environmental accumulation, making them sustainable for fragile ecosystems [254].

These fertilisers lower greenhouse gas emissions and water pollution while reducing labor and input costs due to fewer applications. Future research should optimize designs for diverse crops and conditions, develop cost-effective production, and enhance farmer education through extension services and subsidies [255]. Long-term studies on environmental and health impacts are essential for ensuring safety and sustainability.

### 6.4. Integrated Nutrient Management

Integrated nutrient management (INM) is a holistic strategy that optimizes nutrient availability, enhances soil health, and boosts crop productivity while minimizing environmental harm (Table 4). By combining organic, inorganic, and bio-based fertilisers with agronomic, genetic, and biotechnological approaches, INM improves NUE and reduces losses through leaching, volatilization, and denitrification, supporting sustainable agriculture [256]. Agronomic practices such as precision fertilization, crop rotation, and cover cropping are central to INM, enhancing nutrient cycling and soil health. Precision fertilization delivers nutrients accurately in terms of amount, timing, and location, reducing waste and environmental impact [257]. In addition, crop rotation and cover cropping improve soil structure, increase organic matter content, and minimize nutrient runoff, promoting efficient nutrient use.

Genetic advancements enhance INM by developing crop varieties with superior nutrient uptake and utilisation. For instance, nitrogen-efficient wheat cultivars have shown potential to increase yields while reducing reliance on excessive nitrogen fertilisers [258]. These cultivars thrive in nutrient-limited environments, making them essential for sustainable farming. Biofertilisers, nanofertilisers, and controlled-release fertilisers further improve nutrient delivery. Biofertilisers, such as nitrogen-fixing bacteria enhance natural nutrient cycling and soil fertility [259]. Nanofertilisers and controlled-release fertilisers provide a gradual nutrient release, minimizing over-application and environmental contamination.

The INM also supports sustainable agricultural intensification, increasing food production without expanding farmland, thus preserving ecosystems and biodiversity. As the global population grows, INM is critical for achieving food security while protecting the environment. By integrating traditional knowledge with modern innovations, INM provides a resilient, sustainable framework for agriculture that meets current demands without jeopardizing the needs of future generations [260].

**Table 4:** Effects of advanced fertiliser technologies and integrated nutrient management (INM) on crop productivity.

Crop	Strategy	Effect on Nitrogen Use Efficiency (NUE)	Findings	References
Maize	Controlled-release coated fertilisers	Increased NUE	Reduced N input by ~40% while maintaining yield; significantly reduced TN, NO_3^−^_–N, and TP runoff.	[261]
Maize	Polyurethane-Coated Urea (PCU)	Improved NUE	Maintained yield with 20% less N applied; increased net profit by 8.5–15%; reduced apparent N loss by 36%.	[262]
Wheat	CRF combined with straw return	Improved NUE and economic efficiency	Increased soil N, microbial abundance, root growth, and yield (10–47%); allowed 33% reduction in N application.	[263]
Tomato	Lignin-bentonite nano-coated urea at 25% N rate	NUE increased by 47–88% (vs. 33% in control)	Enhanced growth, yield, and fruit quality with 75% less N; improved firmness and acidity.	[264]
Tomato	Nano-bio phosphorus + *Pseudomonas putida* inoculation	Enhanced P availability and uptake	Improved root/shoot growth, fruit yield, firmness, and vitamin C and flavonoid contents; synergistic effect with bacteria.	[265]
Rice	Nano urea (foliar)	High NUE (80–90%)	Statistically similar growth and yield as conventional urea with 50% less N; efficient nutrient delivery.	[266]
Rice	Optimized N rate (135 kg N ha^−1^)	Improved agronomic efficiency of nitrogen	Sustained yield for 6 years; reduced ammonium N and total N runoff; recommended for sustainable intensification.	[267]
Malt barley	Cover cropping + reduced N fertilization (40 kg N ha^−1^)	Improved soil C sequestration	Enhanced soil organic C in the surface layers; no significant improvement in soil N; sustainable for dryland rotations.	[268]
Potato	Synthetic gel structures with hydrogels + agrochemicals	Reduced leaching, improved retention	Increased yield (6–15 t/ha), saved water (130–200%), reduced pathogen incidence, minimized environmental pollution.	[269]
Maize	Nano-ZnO seed coating (150 mg L^−1^)	Enhanced Zn, Fe, and Mn uptake	Improved growth (5–13%), chlorophyll (141%), photosynthesis; effective in alkaline soils.	[270]
Sorghum	Site-specific N management on marginal lands	Optimized nutrient removal	Achieved comparable yields on marginal lands with adapted N rates (56–112 kg N ha^−1^)	[271]

### 6.5. Circular Nitrogen Economy: Recycling Agricultural Waste as a Nitrogen Source

The linear model of nitrogen use—synthetic fertiliser production, application, and loss—is unsustainable. A circular nitrogen economy offers a sustainable alternative by recycling nitrogen from agricultural waste, such as crop residues, animal manure, and food waste, to create a closed-loop system (Table 5). This approach reduces reliance on synthetic fertilisers, enhances soil health, and mitigates environmental harm. These methods help close the nitrogen loop, reduce eutrophication, and lower greenhouse gas emissions. Despite challenges like high costs, technical barriers, and regulatory constraints, these strategies pave the way for sustainable nitrogen management and resilient agricultural systems.

While organic recycling and circular nitrogen management offer substantial environmental and agronomic benefits, their potential contribution must be evaluated through a mass balance perspective [272]. Globally, modern agriculture relies on more than 100 million tons of nitrogen annually fixed via the Haber–Bosch process to sustain current crop yields. In contrast, the total recoverable nitrogen contained in organic waste streams—including livestock manure, crop residues, food waste, sewage sludge, and agro-industrial by-products—is finite, spatially unevenly distributed, and subject to significant losses during collection, processing, storage, and application. Even under optimistic recovery efficiencies, these streams cannot fully match the magnitude, temporal flexibility, or geographic concentration of synthetic nitrogen inputs required by intensive cropping systems [273].

Moreover, much of the nitrogen present in organic residues is organically bound and released slowly through mineralization, making synchronization with peak crop nitrogen demand challenging [274]. This temporal mismatch can constrain yield potential in high-input systems and increases the risk that recycled nitrogen functions primarily as a background nutrient supply rather than a complete substitute for mineral fertilizers. Logistical limitations—including transport costs, biosecurity concerns, nutrient dilution, and variable nutrient composition—further restrict the scalability of organic nitrogen recycling, particularly in regions with limited livestock density or weak waste management infrastructure [275].

Consequently, organic waste-derived nitrogen should be viewed as a strategically important supplement rather than a wholesale replacement for Haber–Bosch nitrogen at the global scale. Its greatest value lies in partially offsetting synthetic fertiliser demand, improving soil organic matter, enhancing nutrient retention, and closing regional nitrogen loops to reduce environmental losses. When integrated with improved nitrogen use efficiency, precision fertilization, and biologically enhanced systems, organic recycling can significantly reduce reliance on synthetic nitrogen inputs while maintaining productivity.

#### 6.5.1. Recycling Nitrogen from Agricultural Waste

Human activities, particularly the overuse of synthetic fertilisers, lead to eutrophication, greenhouse gas emissions, and soil degradation. A circular nitrogen economy, which emphasizes efficient nitrogen recycling, is essential for sustainable agriculture and environmental protection. Recycling nitrogen from agricultural waste, such as crop residues and animal manure, through processes like composting and anaerobic digestion is a key strategy.

Organic waste gradually transforms into stable humus, releasing plant-available nitrogen (NH_4^+^_ and NO_3^−^_), improving soil structure, enhancing water retention, and inducing microbial activity [276]. Anaerobic digestion (AD) decomposes organic matter in oxygen-free conditions, producing biogas and nutrient-rich digestate containing NH_4^+^_, which serves as an effective fertiliser. The AD is particularly effective for livestock manure, reducing greenhouse gas emissions compared to traditional storage methods [277]. However, high infrastructure costs, technical expertise requirements, and regulatory barriers limit widespread adoption. Future efforts should focus on optimizing these processes, improving cost-effectiveness, and integrating them into farming systems.

#### 6.5.2. Bio-Based Fertilisers

Organic fertilisers, derived from organic sources like animal manure, compost, and digestate from biogas production, offer a sustainable alternative to synthetic fertilisers. These fertilisers release nitrogen gradually in the form of NH_4^+^_ and NO_3^−^_, minimizing losses through leaching and volatilization [278]. For instance, digestate from anaerobic digestion is rich in NH_4^+^_ and other nutrients, enhancing soil organic matter and microbial activity while serving as an effective fertiliser. Studies show that bio-based fertilisers can improve crop yields while reducing greenhouse gas emissions and water pollution compared to synthetic fertilisers [279]. By converting waste into valuable resources, bio-based fertilisers support the circular economy and reduce reliance on energy-intensive synthetic nitrogen production. However, challenges such as inconsistent nutrient content, transportation costs, and regulatory hurdles must be addressed to promote their adoption. With effective management and standardization, bio-based fertilisers can significantly contribute to sustainable agriculture and nitrogen cycling.

#### 6.5.3. Nitrogen Recovery from Wastewater

Recovering nitrogen from wastewater is a vital strategy for achieving a circular nitrogen economy, addressing nutrient pollution and resource scarcity. Advanced technologies, such as NH_3_ stripping and membrane filtration, enable efficient nitrogen recovery from agricultural and municipal wastewater. The NH_3_ stripping converts NH_4^+^_ in wastewater into gaseous NH_3_ that can be captured and transformed into ammonium-based fertilisers. Membrane filtration, including reverse osmosis and electrodialysis, selectively separates nitrogen compounds for reuse in agriculture [280]. These technologies reduce nitrogen pollution in water bodies, mitigating eutrophication, and provide a sustainable nitrogen source for fertiliser production, decreasing reliance on energy-intensive synthetic fertilisers. For example, recovered nitrogen can be used to produce ammonium sulfate or ammonium nitrate for agricultural use [281]. By transforming waste into resources, nitrogen recovery supports the circular economy and reduces greenhouse gas emissions associated with conventional nitrogen production. However, high operational costs, energy demands, and the need for advanced infrastructure pose challenges.

**Table 5:** Effects of circular nitrogen economy on crops.

Crop	Strategy	Effect on Nitrogen Use Efficiency (NUE)	Findings	References
Maize	Enhancing inherent soil productivity	Increased nitrogen partial factor productivity, improved NUE	Enhancing inherent soil productivity beyond 8.0 t/ha increases maize yield by 1.2 t/ha and nitrogen use efficiency. This improvement also reduces nitrogen input and nitrogen losses, supporting more sustainable maize production.	[282]
Wheat	Digestate with nitrification inhibitor	Reduced N losses, comparable yield to mineral fertiliser	Acidified digestate reduced NH_3_ and N_2_O losses; yield comparable to mineral fertilisers	[283]
Tomato	Ammonium–nitrate nitrogen mass ratios in controlled greenhouse conditions	Highest NUE at 75:25 ratio, significantly better fertiliser utilisation	75:25 ammonium-to-nitrate ratio gave highest yield, fertiliser utilisation, N, P, K accumulation, soluble sugars, solids, and vitamin C;. nutrient loss was minimized.	[284]
Rice	Data-driven nutrient management	Enhanced NUE by reducing surplus	Reduced excessive N use by 18 kg/ha without yield loss; increased NUE by 36%	[285]
Soybean	Biochar and biofertilisers	Enhanced nitrogen fixation and soil N content	Biofertilisers improved soybean yield by 5–9%, enhanced biomass and N accumulation	[286]
Barley	Digestate and manure fertilisers	Comparable NUE to mineral fertiliser	Digestate fertilization showed similar/lower N_2_O emissions and maintained yield	[287]
Potato	Compost + mineral fertiliser	Improved NUE and soil nutrients	Yield increased up to 18%, improved soil bulk density and nutrient content	[288]
Maize	Fertiliser types: Farmer Practice (FP), Nutrient Expert (NE), Stable Compound (SF), CRU	Nutrient Expert reduced NH_3_ volatilization but increased N_2_O emission; SF reduced NH_3_, N_2_O, CO_2_, GWP, and increased yield; CRU had greatest NH_3_ reduction with moderate N_2_O increase	SF best balanced emission reductions with highest yield increase (16%); CRU excelled in NH_3_ reduction; NE improved NUE but increased N_2_O emissions	[289]
Wastewater treatment/Nitrogen streams	Integration of electrokinetic processes and air stripping (including innovative air stripping designs and electrochemical cell technologies)	High ammonia removal (~90%) with energy consumption between 5 and 20.4 kWh/kg NH_4^+^_-N; promotes sustainable nitrogen cycle with reduced emissions	Electrokinetic processes enable in situ pH control without chemicals, improving ammonia/ammonium removal and recovery efficiency; hybrid technologies optimize energy use, recovery rate, and co-product valorization	[290]
Cucumber	Combined application of biogas slurry (10, 20, 30 t/ha) and inorganic fertiliser (100%, 75%, 50% recommended dose)	Improved NUE by increasing soil organic matter, nitrogen content, and nutrient availability; potential emission reductions	Biogas slurry increased soil organic matter (0.3% to 2.1%), total N (0.06% to 0.15%), P_2_O_5_ (93 to 224 ppm), and K_2_O; yielded higher fruit number, fruit weight, and total fruit yield; enabled reduction in inorganic fertiliser dose without yield loss	[291]

#### 6.5.4. Environmental, Economic Benefits, and Challenges of Circular Nitrogen Economy

##### Reduced Greenhouse Gas Emissions

Recycling nitrogen from waste significantly lowers greenhouse gas emissions by reducing reliance on synthetic fertilisers produced through the energy-intensive Haber–Bosch method. Synthetic fertiliser production and application are major sources of N_2_O, a greenhouse gas with a global warming potential 265 times that of CO_2_ [292]. By recovering nitrogen from agricultural waste, wastewater, and other organic sources, the circular nitrogen economy minimizes N_2_O emissions and reduces carbon footprint of agriculture. For example, anaerobic digestion and composting recycle nitrogen while also decreasing CH_4_ emissions from decomposing organic waste, contributing to climate change mitigation and sustainable nutrient management.

##### Improved Soil Health

Organic nitrogen sources, such as compost, bio-based fertilisers, and digestate, enhance soil health by increasing organic matter and microbial diversity. These materials improve soil structure, water retention, and nutrient availability, boosting fertility and resilience against erosion and drought [293]. By enhancing soil health, circular nitrogen practices improve agricultural productivity and contribute to long-term environmental sustainability.

##### Economic Savings

The circular nitrogen economy provides economic benefits by lowering input costs for farmers and creating revenue opportunities for industries. Farmers can reduce expenses by using recycled nitrogen sources like compost, digestate, and bio-based fertilisers instead of synthetic fertilisers. Industries can generate income by converting waste into valuable products, such as ammonium sulfate or biogas [294]. For example, anaerobic digestion facilities produce nutrient-rich digestate and biogas, which can be sold as renewable energy. These economic incentives enhance resource efficiency and make circular nitrogen practices financially attractive for sustainable agriculture.

##### Circular Nitrogen Economy Challenges

Despite its environmental and resource efficiency advantages, the implementation of a circular nitrogen economy remains constrained by several interrelated technological, regulatory, and socio-economic challenges.

Technological barriers represent a primary limitation. The large-scale deployment of advanced nitrogen recovery technologies—including ammonia stripping, membrane filtration, and electrochemical nutrient recovery—requires substantial capital investment, specialized infrastructure, and considerable energy inputs [280]. For example, ammonia stripping is associated with high operational costs and demands technical expertise, factors that restrict its adoption, particularly among small- and medium-scale operations. Continued research and development efforts are therefore necessary to optimize process efficiency, lower operational costs, and enhance technological accessibility, especially in developing regions.

Policy and regulatory frameworks also play a decisive role in enabling circular nitrogen systems. Supportive instruments such as subsidies, tax incentives, and research or infrastructure grants can significantly promote nutrient recovery initiatives [276]. The European Union Nitrates Directive, for instance, regulates fertilizer application and encourages the use of organic nutrient sources to mitigate nitrate pollution. However, fragmented policy design, inconsistent implementation, and weak enforcement mechanisms can hinder progress. A coordinated and cross-sectoral approach involving agricultural producers, industry actors, and governmental institutions is therefore essential to establish a stable and enabling regulatory environment.

Farmer adoption constitutes another critical challenge. The transition toward circular nitrogen practices is often limited by insufficient awareness, restricted technical knowledge, and financial constraints. Targeted education and training programs are essential to demonstrate the agronomic, economic, and environmental benefits of recycled nitrogen sources and to support their effective integration into farm management systems [272]. Demonstration projects, advisory services, and farmer cooperatives can further showcase the performance of compost, bio-based fertilizers, and digestate under practical conditions. Strengthening capacity-building initiatives and providing institutional support will be key to facilitating widespread adoption and ensuring the long-term viability of circular nitrogen management strategies.

## 7. Barriers to Implementation and Future Directions

### 7.1. Regional and Policy Challenges

Effective nitrogen sustainability requires region-specific policies tailored to diverse agricultural systems, socio-economic conditions, and environmental contexts. Nitrogen management strategies must address regional disparities, as a one-size-fits-all approach is ineffective. For example, industrialized agriculture in North America and Europe often struggles with excessive synthetic fertiliser use, causing nitrogen runoff and water pollution, while smallholder farms in Sub-Saharan Africa and South Asia face nitrogen deficiencies, limiting crop yields and jeopardizing food security [295]. Policies must balance enhancing productivity with minimizing environmental harm.

Best management practices, such as precision agriculture, crop rotation, and integrated soil fertility management, can optimize nitrogen agronomic efficiency and reduce environmental losses. Regulatory measures, like nitrogen budgeting and fertiliser application caps, help mitigate pollution, as demonstrated by the European Union Nitrates Directive, though its success varies due to inconsistent enforcement across the member states. International cooperation is also critical to address transboundary nitrogen issues, such as eutrophication and atmospheric deposition. A global nitrogen management framework, similar to the Paris Agreement, could promote knowledge sharing and coordinated action, but differing national priorities and capacities pose challenges [121]. Strong governance, stakeholder engagement, and financial incentives are essential to drive effective policy implementation.

### 7.2. Biotechnology Innovations

Biotechnological advances offer transformative solutions for nitrogen management in agriculture. Microbial inoculants, such as *Rhizobium*, enhance BNF by converting atmospheric nitrogen into plant-usable forms. Recent progress in microbial genomics and synthetic biology has engineered robust beneficial strains capable of flourishing under varied environmental stresses, while simultaneously enhancing plant growth via phytohormone secretion and effective pathogen antagonism [296].

Genetic engineering and genome-editing technologies, like CRISPR-Cas9, are being explored to develop nitrogen-fixing cereal crops, such as wheat and maize, which traditionally rely on synthetic fertilisers. Though still in early stages, these innovations could significantly reduce fertiliser dependency and improve NUE. Nanotechnology also shows promise, with nanofertilisers delivering nitrogen in a controlled, targeted manner based on environmental cues like soil moisture or pH, minimizing losses. Nanosensors enable real-time monitoring of soil nitrogen levels, supporting precision agriculture [297]. Despite their potential, biotechnological innovations face challenges, including regulatory barriers, public skepticism, and high development costs. Long-term environmental and health impacts require thorough assessment to ensure sustainability.

### 7.3. Climate Change Resilience

Climate change complicates nitrogen sustainability by altering nitrogen cycling through rising temperatures, shifting precipitation patterns, and extreme weather events. Higher temperatures increase ammonia volatilization and nitrate denitrification, leading to greater nitrogen losses and greenhouse gas emissions. Droughts and floods disrupt nitrogen availability, reducing crop yields and threatening food security [175]. Climate-resilient nitrogen management strategies are essential to sustain agricultural systems in a warming world.

Climate-smart practices, such as conservation tillage, cover cropping, and agroforestry, enhance NUE while improving soil health and water retention, reducing vulnerability to climate stressors. Slow-release fertilisers and nitrification inhibitors align nitrogen availability with crop needs, minimizing losses during extreme weather. Breeding climate-resilient crop varieties with enhanced nitrogen uptake and assimilation is another key strategy. For instance, wheat varieties with robust root systems access deeper soil nitrogen during droughts, while heat-tolerant rice maintains yields under high temperatures [298].

## 8. Conclusions

This review elucidates innovative strategies for enhancing NUE, integrating plant–soil dynamics with biotechnological, precision agriculture, and circular economy approaches to address inefficiencies and environmental impacts in agriculture. By synthesizing recent advancements in genetic editing, microbial inoculants, and smart fertilisers, it underscores the potential to reduce reliance on synthetic fertilisers, mitigate greenhouse gas emissions, and bolster climate resilience—critical for global food security amid a growing population and changing climate. However, limitations include high implementation costs, regulatory hurdles, and the need for long-term field validations across diverse ecosystems. Future work should prioritize interdisciplinary research, scalable technologies, and policy frameworks to promote widespread adoption. Ultimately, these strategies pave the way for sustainable nitrogen management, ensuring productive agroecosystems while preserving environmental integrity for future generations.

## Data Availability

Data is contained within the article.

## Abbreviations

The following abbreviations are used in this manuscript:

AMT1	Ammonium transporter 1
AMF	Arbuscular mycorrhizal fungi
AI	Artificial intelligence
BNF	Biological nitrogen fixation
CEC	Cation exchange capacity
DNRA	Dissimilatory nitrate reduction to ammonium
GIS	Geographic Information Systems
GOGAT	Glutamate synthase
GS	Glutamine synthetase
MAS	Marker-assisted selection
PM2.5	Particulate matter
PGPR	Plant growth-promoting rhizobacteria
QTLs	Quantitative trait loci
ROS	Reactive oxygen species
